# How to Employ Trained Immunity and Trained Immunity-Based Vaccines to Inhibit Allergic Inflammation

**DOI:** 10.3390/vaccines14030268

**Published:** 2026-03-16

**Authors:** Wonho Kim, Dooil Jeoung

**Affiliations:** Department of Biochemistry, Kangwon National University, Chuncheon 24341, Republic of Korea; kimwonho99@kangwon.ac.kr

**Keywords:** allergen specific immunotherapy, immune tolerance, innate cells, trained immunity, trained immunity-based vaccines

## Abstract

Trained immunity confers protection against subsequent unrelated infections through metabolic and epigenetic reprogramming. Unlike adaptive immunity, trained innate immunity provides broad, non-specific protection against diverse heterologous pathogens. In addition to potentiating inflammatory responses upon secondary challenge, trained innate immune cells can also acquire anti-inflammatory and tolerogenic phenotypes, a property with important implications for chronic inflammatory diseases such as allergic disorders. Trained immunity-based vaccines (TIbVs) have emerged as promising immunomodulatory strategies capable of attenuating allergic inflammation by inducing immune tolerance. Similarly, allergen-specific immunotherapy (AIT) promotes long-term tolerance to allergens through metabolic and epigenetic reprogramming of innate immune cells. AIT drives the differentiation of monocytes into tolerogenic dendritic cells, thereby reshaping downstream adaptive immune responses. This review summarizes the current understanding of trained immunity and its role in protection against the same and heterologous infections. We discuss the molecular mechanisms underlying trained immunity, with an emphasis on metabolic and epigenetic reprogramming. Furthermore, we highlight the therapeutic potential of TIbVs and AIT as next-generation vaccines for allergic diseases. A deeper understanding of AIT-induced immune tolerance, the identification of predictive biomarkers, and the optimization of delivery platforms—such as lipid nanoparticle-based systems—will be critical for improving the safety and efficacy of future anti-allergy vaccines.

## 1. Introduction

Trained immunity refers to the capacity of innate immune cells to undergo functional reprogramming following an initial stimulus, resulting in enhanced or altered responses to subsequent challenges, whether homologous or heterologous in nature [1,2,3]. Unlike adaptive immunity, which relies on antigen-specific memory mediated by B and T lymphocytes, trained immunity operates independently of antigen specificity and is driven by sustained metabolic and epigenetic modifications. In addition to heightened inflammatory responsiveness, trained immunity can also induce tolerogenic phenotypes that limit excessive inflammation and tissue damage, thereby contributing to immune homeostasis. Trained immunity has been observed in multiple innate immune cell types, including monocytes, dendritic cells (DCs), macrophages, and natural killer (NK) cells [4]. Increasing evidence further suggests that non-hematopoietic cells, such as epithelial cells, can also acquire trained immunity–like features [5]. In Rag1-deficient mouse models, intranasal administration of the inactivated polybacterial mucosal vaccine MV130 confers protection against viral respiratory infections through trained immunity induced in bone marrow progenitors and circulating monocytes [6]. Thus, innate immune memory can develop in the absence of T and B cells.

*Candida dubliniensis* infection promotes bone marrow expansion and myelopoiesis, leading to the generation of myeloid-derived suppressor cells (MDSCs) that protect against acute polymicrobial sepsis [2]. This protective response is accompanied by increased production of granulocyte colony-stimulating factor (G-CSF), CXCL2, and CCL2, highlighting the role of cytokine-driven myeloid reprogramming in trained immunity [2]. Similarly, CL429, a synthetic agonist of Toll-like receptor 2 (TLR2) and nucleotide-binding oligomerization domain 2 (NOD2), protects mice from leptospirosis by inducing trained immunity in peritoneal macrophages independently of adaptive immune responses [7]. Collectively, these studies indicate that trained immunity can be established at both mature innate immune cell and progenitor cell levels, enabling long-term functional memory.

In this review, we summarize current knowledge of the mechanisms underlying trained immunity, with particular emphasis on metabolic and epigenetic reprogramming. We also review recent advances in allergen-specific immunotherapy (AIT) and discuss the emerging concept of trained immunity-based vaccines (TIbVs) as potential therapeutic approaches for chronic inflammatory diseases, including allergic disorders.

## 2. Characteristics of Trained Immunity

Trained immunity is defined by several distinguishing features that differentiate it from classical adaptive immune memory. First, trained immunity is antigen-independent and non-specific, enabling innate cells to respond more robustly to a broad range of secondary stimuli rather than to a single cognate antigen [8,9]. Second, trained immunity is mediated by epigenetic and metabolic reprogramming rather than somatic recombination involving antibodies and T cell receptors (TCRs), which are hallmarks of adaptive immunity. These molecular changes persist for weeks to months and underlie the long-term functional adaptation of innate immune cells. Thirdly, the nonspecific protection against heterologous infections following induction of trained immunity can, in some contexts, be transmitted to offspring via epigenetic reprogramming [10].

Bacillus Calmette-Guérin (BCG) vaccine can reprogram hematopoietic stem and progenitor cells with imprinting in innate immune cells [11,12]. Signals originating from peripheral immune activation can reach the bone marrow and induce durable reprogramming of hematopoietic stem and progenitor cells (HSPCs), thereby biasing myelopoiesis to functionally trained innate immune cells. This progenitor-level reprogramming provides a mechanistic basis for the long-lasting nature of trained immunity, even in the absence of long-lived mature innate immune cells. The fact that trained circulating monocytes are present in BCG-vaccinated individuals for at least several months suggests that functional reprogramming takes place at the level of myeloid progenitor cells in the bone marrow [13]. Thus, trained immunity occurs in mature myeloid cells, but its effects are also initiated at the level of precursor cells of the innate immune system in the bone marrow, leading to long-lasting responsiveness.

During trained immunity, an initial stimulus induces long- term epigenetic and metabolic changes that render the cells more responsive to secondary stimulation. Trained immunity displays the persistence of its epigenetic changes after the initial stimulus has ceased and a return to baseline cellular activation status [14]. Hypomethylation at pro-inflammatory gene loci, can be a key epigenetic mechanism contributing to long-term trained immune memory (TRIM). These hypomethylated states are stable and faithfully inherited through cell division. This DNA-methylation-mediated process includes hematopoietic stem cell self-renewal, differentiation from central to peripheral compartments, and autonomy of tissue-resident cells [15,16].

A defining hallmark of trained immunity is the enhanced production of pro-inflammatory cytokines, such as tumor necrosis factor (TNF), interleukin-6 (IL-6), and interleukin-1β (IL-1β), following secondary stimulation [17]. Increased chromatin accessibility at promoter and enhancer regions facilitates transcription of genes encoding pro-inflammatory cytokines, leading to elevated cytokine expression. This enhanced production of pro-inflammatory cytokines via DNA hypomethylation can enhance pathogen clearance.

Trained immunity can also manifest as immune tolerance, characterized by attenuated pro-inflammatory cytokine responses following repeated or prolonged exposure to specific stimuli. This tolerogenic form of trained immunity plays a critical role in preventing excessive inflammation and tissue damage. For example, exposure to lipopolysaccharide (LPS) can induce endotoxin tolerance in monocytes and macrophages, resulting in diminished pro-inflammatory cytokine production while preserving antimicrobial functions [18,19]. LPS exposure activates macrophages through TLR4 signaling, inducing antimicrobial innate immune responses [20]. BCG and beta-glucan induce the differentiation of monocytes into DCs, which inhibits pro-inflammatory T cell response [21]. BCG and β-glucan induce trained immunity by epigenetically and metabolically reprogramming monocytes and macrophages, leading to decreased pro-inflammatory cytokine responses [21]. An adenovirus-vectored Rv2299c vaccine enhances protection against tuberculosis by inducing trained immunity in DCs [22].

Innate immune cells, such as mononuclear macrophages, NK cells, and DCs, undergo molecular and functional reprogramming after encountering stimuli [23,24]. This reprogramming is reversible and context-dependent, allowing innate immune cells to adapt to varying environmental cues. The β-glucans from *Saccharomyces cerevisiae* induce trained immunity in monocytes by activating TLR4 and MMR receptors [25]. *Candida albicans* induces trained immunity in neutrophils, which in turn clears *Candida albicans* via mitochondrial ROS production [26]. The BCG vaccine protects against lethal candidiasis in severe combined immunodeficiency (SCID) mice, by inducing trained immunity in monocytes via TLR4 and NOD2 receptor [27]. The BCG vaccine can induce trained immunity and provides protection against unrelated pathogens (e.g., against HIV and TB) [28]. BCG administration in healthy adults induces trained immunity in monocytes and NK cells to confer protection against mycobacteria and other unrelated pathogens, such as *Staphylococcus aureus* or *Candida albicans* via IFN-γ and IL-1β in response to *Staphylococcus aureus* stimulation [29]. BCG vaccination can enhance innate immune responses by increasing levels of pro-inflammatory cytokines such as IL-18, tumor necrosis factor-α (TNF-α), tumor necrosis factor super family -10 (TNFSF-10), and vascular endothelial growth factor A (VEGFA), which might support antiviral defense, although its protective effect against COVID-19 remains under investigation [30]. Pro-inflammatory cytokines such as IL-1, IL-6 and IL-8 can help eliminate the pathogens by recruiting leukocytes to the site of infection [31].

**Figure 1:**
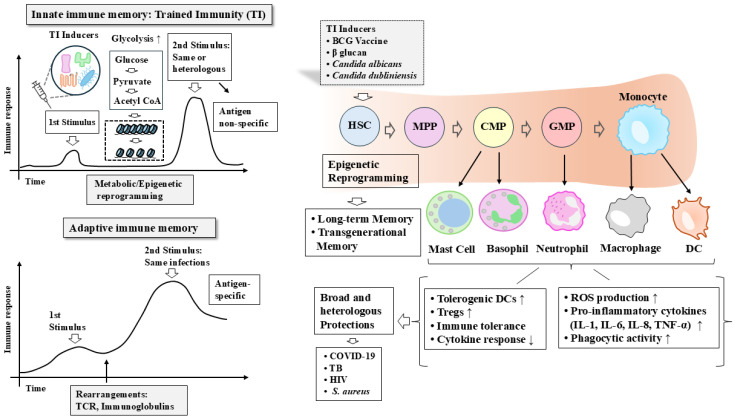
Trained immunity provides enhanced protection against both homologous and heterologous infection. Trained immunity occurs not only in hematopoietic stem cells (HSCs) but also in innate immune cells, including monocytes, dendritic cells, neutrophils, and NK cells, as well as in various non-immune cells. After the initial stimulus is removed, innate immune cells undergo epigenetic and metabolic reprogramming, enabling enhanced protection upon secondary stimulation by the same or different pathogens. In contrast, adaptive immunity relies on gene rearrangements involving T cell receptors and immunoglobulins. Trained immunity induces epigenetic reprogramming in HSCs and is characterized by non-specific responses rather than antigen specificity. Key features of trained immunity are shown. HSC, hematopoietic stem cell; MPP, multipotent progenitor; CMP, common myeloid progenitor; GMP, granulocyte–monocyte progenitor. The hollow arrow and thick arrow denote the direction of reaction. ↑ denotes an increase in expression or activity, whereas ↓ denotes a decrease in expression or activity.

Collectively, these characteristics highlight trained immunity as a dynamic and adaptable form of innate immune memory. By integrating metabolic and epigenetic reprogramming across multiple cell types, trained immunity provides a versatile mechanism for host defense while maintaining immune homeostasis. Understanding these defining features is essential for harnessing trained immunity in the development of novel immunotherapeutic strategies. The differences between adaptive immunity and trained immunity, along with the key features of trained immunity, are illustrated (Figure 1).

## 3. Molecular Mechanisms of Trained Immunity

Microbial ligands that bind specific pattern recognition receptors (PRRs) on myeloid cells can induce persistent metabolic and epigenetic reprogramming in innate immune cells [32,33]. *Candida albicans* infection in Rag2-/- mice leads to protection against reinfection via epigenetic (H3K4me3) and metabolic reprogramming of monocytes by β-glucan [34]. Specific microbial products, such as those derived from certain helminths can induce an anti-inflammatory trained immunity phenotype via oxidative phosphorylation (OXPHOS), leading to the production of high levels of anti-inflammatory cytokines such as IL-10 or IL-1RA [35]. It is thus probable that trained immunity can induce immune tolerance to secondary infections.

The establishment of trained immunity is driven by coordinated metabolic and epigenetic reprogramming, enabling innate immune cells to respond more rapidly and robustly to secondary stimuli and thereby providing the mechanistic basis for innate immune memory. Monocytes are differentiated into macrophages by *β*-glucan via epigenetic and metabolic reprogramming [36]. These changes prime macrophages to exhibit enhanced functional activity, such as improved phagocytic capacity and higher reactive oxygen species (ROS)/nitric oxide (NO) production [36]. The trained immunity phenotype of monocyte-derived macrophages increases the production of pro-inflammatory cytokines such as TNF-α, interleukin (IL)-6, and IL-1β upon secondary stimulation [37]. These studies suggest that innate immune reprogramming can lead to either enhanced inflammatory responses or immune tolerance, depending on the nature and context of the stimulus.

### 3.1. Metabolic Reprogramming in Trained Immunity

BCG-derived outer membrane vesicles enhance trained immunity in hematopoietic stem cells via TLR2-dependent activation of aerobic glycolysis [12]. Innate immune cells shift from OXPHOS to aerobic glycolysis (the Warburg effect), providing energy for enhanced effector function [13]. Enhanced aerobic glycolysis is fundamentally linked to the induction of trained immunity [38], supporting the long-term, heightened responsiveness of innate immune cells, including microglia. Trained immunity is induced by immunological signaling and metabolic reprogramming mediated by hypoxia-inducible factor 1-α (HIF1-α) downstream of mammalian target of rapamycin (mTOR) [39]. *Mycobacterium tuberculosis* antigen Rv1471induces innate immune memory in macrophages by enhancing aerobic glycolysis via AKT-mTOR-HIF-1α signaling axis [40]. HIF-1α promotes the expression of glycolytic enzymes, including 6-phosphofructo-2-kinase/fructose-2,6-bisphosphatase 3 (PFKFB3), thereby enhancing glycolysis and increasing lactate production, which in turn contributes to the activation of histone-modifying enzymes and epigenetic reprogramming [41]. Acetylation of PFKFB3 at lysine promotes glycolysis [42]. PFKFB3-driven glycolysis increases lactate levels, which induce histone lactylation (H4K12la) at the promoters of target genes, leading to elevated expression of pro-inflammatory mediators such as IL-6 and matrix metalloproteinase-9 (MMP-9) [43]. These reports highlight a tight link between metabolic and epigenetic reprogramming in trained immunity.

### 3.2. Epigenetic Reprogramming in Trained Immunity

**Figure 2:**
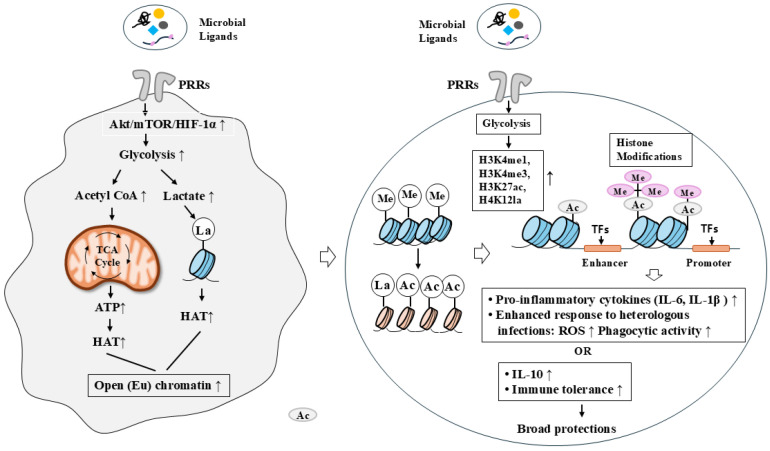
Trained immunity induces metabolic and epigenetic reprogramming in innate immune cells after primary stimulation. Binding microbial ligands to pattern recognition receptors (PRRs) induces metabolic and epigenetic reprogramming in innate immune cells, thereby enhancing responses to secondary infections or promoting immune tolerance. Epigenetic reprogramming facilitates the differentiation of monocytes into macrophages, which subsequently exhibit enhanced responses to secondary infections through increased reactive oxygen species (ROS) production and elevated expression of IL-6 and IL-1β. Engagement of PRRs by microbial ligands activates the AKT/mTOR/HIF-1α signaling pathway, leading to increased glycolytic activity via upregulation of 6-phosphofructo-2-kinase/fructose-2,6-bisphosphatase 3 (PFKFB3). Enhanced glycolysis results in increased lactate production, which induces histone lactylation and promotes the expression of pro-inflammatory cytokines through epigenetic modifications, including the deposition of H3K4me1, H3K4me3, and H3K27ac at the promoters of pro-inflammatory cytokine genes. In addition, microbial ligands can increase the expression of anti-inflammatory cytokines such as IL-10, thereby contributing to the induction of immune tolerance. The hollow arrow and thick arrow denote the direction of reaction. ↑ denotes an increase in expression or activity. Ac, acetylation; HAT, histone acetyl transferase; La, lactylation.

Epigenetic modifications play a pivotal role in trained immunity by establishing long-lasting changes in chromatin accessibility. Trained innate immune cells exhibit enrichment of activating histone modifications, such as H3K4me3 at gene promoters and H3K27ac at enhancers, particularly at loci encoding pro-inflammatory cytokines and pattern recognition receptors [44,45]. The regulation of monocyte-mediated cytokine response in trained immunity is accompanied by the accumulation of epigenetic chromatin marks, such as H3K4me1, H3K4me3, or H3K27 acetylation (H3K27ac), on the promoters and enhancers of the genes encoding for the monocyte-derived cytokines TNF-α, IL-6, and IL-1β [46,47]. These epigenetic marks establish a “poised” chromatin state that enables rapid transcriptional activation upon secondary challenge. The mechanisms underlying trained immunity, which involve metabolic and epigenetic reprogramming, are shown (Figure 2).

## 4. Epigenetic and Metabolic Reprogramming in Allergic Inflammation

### 4.1. The Mechanisms of Allergic Inflammation

Allergic diseases are chronic inflammatory disorders characterized by dysregulated immune responses to otherwise harmless environmental antigens. Innate immune cells, including monocytes, macrophages, dendritic cells, and epithelial cells, are the first to encounter allergens at barrier sites such as the skin, airways, and gastrointestinal tract. Some studies have reported excessive innate immune responses at birth in cord blood- derived mononuclear cells from allergic children compared to non-allergic donors [48]. These responses are characterized by high levels of IL-6, IL-1β and TNF-α following in vitro TLR stimulation [48]. Thus, a predisposition to allergen-specific type 2 responses is linked to early-life activation or imprinting of innate immune cells during the perinatal period. Egg- allergic pediatric patients display high numbers of circulating monocytes and DCs [49]. These reports indicate the roles of innate immune cells in allergic inflammation.

The immunological mechanisms underlying allergies can be divided into two phases: sensitization/memory and effector phase. Sensitization occurs upon first exposure to an allergen and results in the generation of allergen-specific CD4^+^ Th2 cells and allergen-specific IgE antibodies produced by B cells [50]. Allergen-specific IgE binds to the high-affinity FcεRI receptor on mast cells and basophils, establishing the sensitized state of these cells [51]. Recurrent exposure to the same allergen triggers mast cells and basophils to release inflammatory mediators, including histamine and leukotrienes. Cytokines produced by Th2 cells—such as IL-4, IL-5, IL-9, and IL-13—together with type 2 innate lymphoid cells (ILC2s) activated by epithelial alarmins (TSLP, IL-33, or IL-25), contribute to eosinophilia, mucus production, recruitment of inflammatory cells, and tissue inflammation [52]. Epithelial alarmins are increased when allergens activate PRRs [53]. Allergens can damage the epithelium, increasing permeability by disrupting tight junctions. NOD-like receptor family pyrin domain-containing 3 (NLRP3) contributes to maintaining epithelial barrier integrity [54]. These reports indicate that both innate and adaptive immune responses contribute to the pathogenesis of allergic inflammation.

Tolerogenic training can limit excessive inflammatory responses by enhancing the production of anti-inflammatory mediators, such as interleukin-10 (IL-10). Infection with *Nippostrongylus brasiliensis* or exposure to papain allergen induces IL-33 production by epithelial cells, leading to activation and proliferation of lung ILC2s [55,56,57]. ILC2s can be converted into IL-10–producing ILC2s in response to IL-2 and IL-33, playing a critical role in restoring epithelial integrity, suppressing type 2 responses, and promoting tolerance to aeroallergens [58,59]. Thus, plasticity in ILC2 might be linked to epigenetic reprogramming and can be employed for developing anti-allergy drugs.

### 4.2. Epigenetic and Metabolic Reprogramming in Allergic Inflammation

IgE-mediated cow’s milk allergy is associated with decreased H3/H4 acetylation at Treg loci, reflecting an epigenetic state that favors reduced gene expression in Treg cells (Tregs) [60]. IgE-mediated cow’s milk allergy increases the histone acetylation at the signal transducer and activator of transcription 6 (STAT6), a proallergic gene [60]. Histone deacetylases (HDACs) influence airway remodeling [61], and macrophage polarization [62,63]. The inhibition of class IIa, such as HDAC9 promotes M2 macrophage polarization [63]. Since M2 macrophage polarization is essential for allergic inflammation [64], it is thus reasonable that epigenetic reprogramming plays a critical role in allergic inflammation. HDAC1, increased by allergen exposure, increases the expression of Th2 cytokines while decreasing Th1/Th17 cells [65]. Thus, targeting HDACs can provide clues for developing anti-allergy drugs.

Histone demethylases KDM5A, KDM5B, KDM5C, and KDM5D regulate gene expression by demethylating histone H3 at lysine 4 (H3K4) [66]. In a mouse model of allergic airway inflammation, USP7-mediated deubiquitination stabilizes KDM5A, which in turn promotes Th2 cytokine production (e.g., IL-4) via activation of STAT6 and GATA3 signaling [67]. In a mouse model, respiratory syncytial virus (RSV) infection induces KDM5B, which suppresses the expression of type I IFN, IL-6, and TNF-α while promoting the expression of Th2 cytokines [68]. Thus, KDM5B can mediate allergic inflammation. KDM5C can decrease the expression of IL-6, an inhibitor of Treg differentiation, in plasmacytoid dendritic cells (pDCs) in a mouse model of wound healing via IFN-I/TYK2/JAK1,3 signaling pathway [69]. Thus, targeting KDM5C might exert anti-allergic effects. miR-155-5p induces M1 polarization of macrophages by targeting KDM5D in a rat model of liver transplantation [70]. This suggests that KDM5D can contribute to the pathogenesis of allergic inflammation by promoting M2 macrophage polarization. Thus, KDM5D may contribute to allergic inflammation by promoting M2 macrophage polarization. Taken together, these reports indicate the role of epigenetic reprogramming in allergic inflammation.

Allergen binding to IgE on FcεRI activates mast cells, enhancing glycolytic activity and triggering degranulation with the release of inflammatory mediators [71]. Formaldehyde exposure increases lactate production and lactate dehydrogenase (LDH) activity in a mouse model of allergic asthma [72]. Thus, targeting glycolysis can attenuate allergic asthma. Allergic inflammation enhances Th2 cytokine production, including IL-4, IL-5, and IL-13 through glycolysis [73]. ILC2s, key players in type 2 immunity and allergic airway inflammation [74], depend on tightly regulated glycolysis to support their functions and tissue residence [75]. Inhibition of glycolysis by 2-deoxyglucose (2-DG) suppresses allergic airway inflammation by blocking ILC2-mediated upregulation of HIF-1α [76]. These reports imply that coupling between glycolytic activity and epigenetic reprogramming contributes to the pathogenesis of allergic inflammation.

Chromatin remodeling complexes, such as SWI/SNF (BAF) complexes, use ATP—a product of glycolysis—to reposition, eject, or restructure nucleosomes [77]. This suggests that glycolytic activity can modulate chromatin remodeling, thereby affecting the expression of inflammation-related genes during allergic inflammation. Deacetylation of transaldolase 1 (TALDO1) by histone deacetylase 6 (HDAC6) enhances glycolysis by upregulating the expression of hexokinase 2 (HK2) and lactate dehydrogenase A (LDHA) [78]. Antiviral trained immunity in shrimp involves acetylation at H3K27 mediated by enhanced glycolytic activity [79]. Raw milk attenuates allergic inflammation by reducing histone acetylation of Th2 cell-related genes [80]. These reports indicate a close relationship between glycolysis and epigenetic reprogramming.

**Figure 3:**
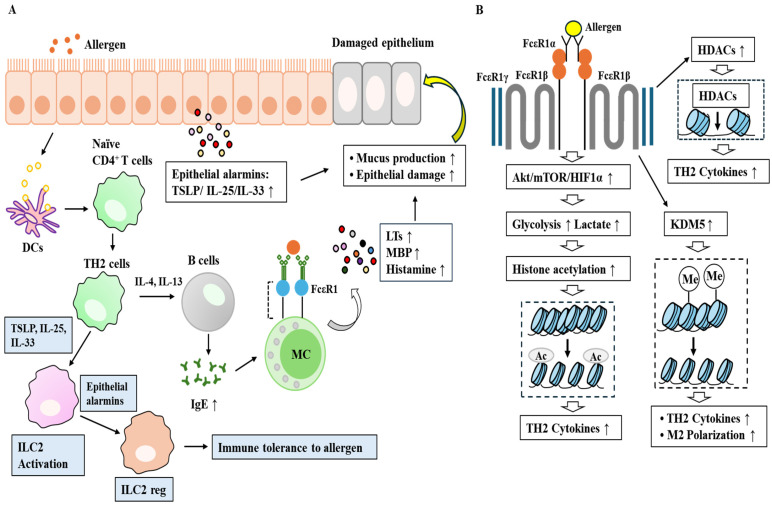
Allergic inflammation is driven by mechanisms involving metabolic and epigenetic reprogramming of immune cells. (**A**) Allergic responses are initiated by the uptake and processing of allergens by dendritic cells (DCs). Allergen-derived peptides drive the differentiation of naïve T cells into T helper 2 (Th2) cells, which promote the production of allergen-specific IgE by B cells through the secretion of IL-4 and IL-13. IgE subsequently binds to FcεRI expressed on mast cells, basophils, and eosinophils. Upon re-exposure to the same allergen, these cells release inflammatory mediators such as histamine, leukotrienes, and myelin basic protein. Th2 cells, in cooperation with group 2 innate lymphoid cells (ILC2s), enhance the production of Th2 cytokines, including IL-4, IL-5, and IL-13. Similar to epithelial cells, Th2 cells produce epithelial alarmins such as TSLP, IL-33, and IL-25, which activate ILC2s during allergic inflammation. Crosstalk between Th2 cells and ILC2s drives hallmark allergic responses, including mucus secretion, eosinophilia, and inflammatory cell recruitment. Notably, ILC2s can be converted into IL-10–producing regulatory ILC2s by epithelial alarmins, thereby suppressing type 2 immune responses and promoting tolerance to allergens. LTs, leukotrienes; MBP, myelin basic protein. (**B**) Allergic inflammation enhances glycolytic metabolism, leading to increased production of Th2 cytokines such as IL-4, IL-5, and IL-13. Glycolysis-induced histone lactylation is required for histamine release and augmented Th2 cytokine production. Allergic inflammation also upregulates histone deacetylases HDAC1 and HDAC3, which suppress Th1 cytokine expression while promoting Th2 cytokine expression. KDM5A enhances Th2 cytokine expression via STAT3 signaling, whereas KDM5B and KDM5C suppress the expression of Th1 cytokines such as IL-6, IFN, and TNF-α. In addition, KDM5D contributes to allergic inflammation by promoting M2 macrophage polarization. The hollow arrow and thick arrow denote the direction of reaction. ↑ denotes an increase in expression or activity.

DNA methylation and histone modifications induced by environmental exposures, such as pollutants, parasites, and tobacco smoke, can reprogram the immune system, leading to increased production of Th2 cytokines, including IL-4 and IL-13, and contributing to the development of allergic diseases [81]. Histone lactylation resulting from enhanced glycolysis is necessary for histamine release and the increased production of Th2 cytokines during allergic inflammation [82]. Upregulation of the glycolytic enzyme enolase 1 (ENO1) increases lactate levels and promotes histone lactylation (H4K8la), thereby coupling glycolysis with epigenetic reprogramming [83]. Lactate can be converted to acetyl-CoA, which can then be used by histone acetyltransferases (HATs) to modify histones. Collectively, these findings confirm that metabolic and epigenetic reprogramming play critical roles in the pathogenesis of allergic inflammation. The mechanisms underlying allergic inflammation involving metabolic and epigenetic reprogramming are illustrated (Figure 3).

## 5. Trained Immunity-Based Vaccines

Trained immunity-based vaccines (TIbVs) are a novel class of immunomodulatory vaccines that harness the ability of innate immune cells to develop long-term functional memory. TIbVs consist of trained immunity inducers and specific antigens. Trained immunity-based vaccines (TIbV) can induce innate immune memory to confer heterologous protection against a broad range of pathogens [84,85,86]. Unlike conventional vaccines, TIbVs provide non-specific protection against emerging viruses or recurring infections for which specific vaccines are unavailable, using components such as BCG, β-glucans, or bacterial extracts [87]. Trained innate immune cells can acquire anti-inflammatory or tolerogenic phenotypes depending on the nature, dose, and duration of the stimulus, the type of TIbV, or the specific pathological context. TIbVs can reduce mortality associated with influenza [88], bacterial sepsis [89], and other bacterial infections [29] by priming innate immune cells to mount amplified cytokine responses.

By promoting tolerogenic trained immunity, TIbVs may attenuate excessive inflammatory responses and restore immune balance. Incorporating “tolerance inducers” such as the BCG vaccine, certain polybacterial preparations, cell lysates, or specific cannabinoids, along with specific allergens can promote both allergen-specific tolerance and broader immune responses [90]. Specific probiotics can prevent severe food allergies by inducing metabolic and epigenetic reprogramming, thereby conferring tolerance to allergens [91]. OM-85, bacterial cell lysates can prevent allergic airway hypersensitivity by increasing regulatory T cell (Treg) numbers in an interleukin-10 (IL-10)-dependent manner and by suppressing the activation of ILC2 [92]. Thus, targeting ILCs may produce effective TIbVs. Innate immune cells trained with specific stimuli can acquire a tolerogenic phenotype, which may have important implications for chronic inflammatory diseases such as allergies [93]. These reports indicate that TIbVs can be developed as anti-allergy therapeutics. Furthermore, the identification and characterization of new tolerance-inducing agents may facilitate the prevention and treatment of allergic diseases.

Trained immunity induced by *Enterococcus faecalis* ribosomal protein S11 (RPS11), delivered via nanoparticles, enhances antigen presentation and improves influenza vaccine efficacy by activating TLR4 signaling [88]. This suggests that lipid nanoparticle (LNP)-based delivery of trained immunity inducers (TIs) could provide broad protection against heterologous infections. Certain nanomaterials, such as pristine graphene, can amplify macrophage inflammatory responses to TLR agonists [94]. Host–pathogen hybrid glycocalyx-mimicking lipid nanoparticles (LNPs) induce trained immunity, providing protection against sepsis [89]. The adjuvant properties of LNPs can enhance the immunogenicity of the vaccine [95]. Thus, LNPs can be employed for developing effective trained immunity-based vaccines. To enhance the efficacy of LNPs in inducing trained immunity, it is necessary to incorporate trained TIs such as pathogen-associated molecular patterns (PAMPs) or damage-associated molecular patterns (DAMPs), and to optimize delivery for targeting key immune cells, including macrophages and dendritic cells (DCs) [96]. The addition of specific immune-stimulating molecules, such as TLR agonists (e.g., monophosphoryl lipid A for TLR4 or CpG motifs for TLR9), can enhance the LNP’s ability to induce immune tolerance in a mouse model of cardiomyopathy [18]. TLR ligand induces trained immunity in T cell and B cell-deficient rag1-/- zebra fish, which confers protection against bacterial infections [97].

Nanoparticles can efficiently target various myeloid cells, making them ideal platforms for the targeted delivery of trained immunity inducers (TIs) [98]. Engineering lipid nanoparticles (LNPs) for targeted delivery to specific immune cells, particularly dendritic cells (DCs) and macrophages in lymphoid organs such as the spleen and bone marrow, is essential for efficient induction of trained immunity [99,100]. Phospholipids, such as lyso-phosphatidylserine (lyso-PS), can influence fusogenicity, cellular uptake, and subsequent immunomodulatory effects, potentially enhancing the delivery of LNPs to specific immune cells [101,102].

Particle size and surface molecules (e.g., phosphatidylserine (PS) or its derivatives) can affect cellular uptake and influence whether the immune response is immunogenic or tolerogenic [103]. Smaller nanoparticles (typically <100 nm) are primarily internalized via endocytosis, whereas larger particles are preferentially phagocytosed by macrophages [104]. When nanoparticles mimic phosphatidylserine (PS) display, they can induce immune tolerance by promoting anti-inflammatory responses in macrophages through activation of indoleamine 2,3-dioxygenase (IDO1) [105]. PS can enhance immune tolerance by promoting the secretion of anti-inflammatory cytokines, such as IL-10 and TGF-β, from B cells, T cells, and NK cells [106]. Lyso-PS can induce oral tolerance by enhancing nanoparticle stability and promoting uptake by innate immune cells in the gut and Peyer’s patches [107]. Manipulating cholesterol content can enhance trained innate immunity, leading to antiviral effects by upregulating low-density lipoprotein receptor (LDLR) expression and type I interferon (IFN) production in macrophages [108]. Since cholesterol accumulation in myeloid cells can induce trained immunity, adjusting cholesterol content may help regulate this process.

**Figure 4:**
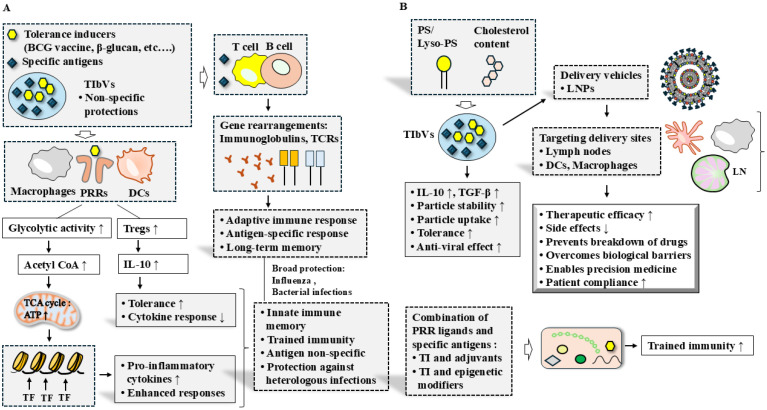
Trained immunity-based vaccines. (**A**) TIbVs provide broad protection by activating both non-specific and antigen-specific immune responses. Trained immunity inducers, such as PRR ligands, stimulate trained immunity through enhanced glycolysis, epigenetic reprogramming, and increased production of pro-inflammatory cytokines, including IL-1β, IL-6, and TNF-α. TIbVs can confer broad, non-specific protection against heterologous pathogens while simultaneously inducing allergen-specific immune tolerance. In parallel, specific antigens included in TIbVs elicit adaptive immune responses. The hollow arrow and thick arrow denote the direction of reaction. ↑ denotes an increase in expression or activity. ↓ denotes an increase in expression or activity. (**B**) Optimization of adjuvants and trained immunity inducers, development of advanced delivery systems such as lipid nanoparticles (LNPs), and combinatorial strategies involving trained immunity inducers and epigenetic modulators may further enhance the efficacy of TIbVs. Targeted delivery of TIbVs to innate immune cells, including DCs and macrophages, can improve vaccine effectiveness. Additionally, modulation of particle size, phospholipid composition, and cholesterol content may enhance both the safety and efficacy of TIbVs. The hollow arrow and thick arrow denote the direction of reaction. ↑ denotes an increase in expression or activity. ↓ denotes an increase in expression. LN, lymph node.

Since TIbVs can induce anti-inflammatory and tolerogenic effects, they hold potential for development as anti-allergy therapeutics. It is necessary to identify novel adjuvants that can enhance both trained immunity and immune tolerance. It is also advisable to develop mucosal delivery systems to confer protection against common infections and allergic inflammation. Excessive or dysregulated induction of trained immunity may exacerbate inflammation, emphasizing the importance of carefully optimizing TIbV design. Advances in vaccine formulations and delivery platforms, including nanoparticle-based systems, provide new opportunities to fine-tune trained immunity responses and enhance safety profiles. Harnessing trained immunity through TIbVs holds significant promise for the prevention and treatment of allergic and other chronic inflammatory diseases. The roles of TIbVs in broad protection and strategies to enhance their therapeutic potential are depicted (Figure 4).

## 6. Allergen Specific Immunotherapy

### 6.1. AIT Can Induce Immune Tolerance

Employing trained immunity to attenuate allergic inflammation is an emerging area of medical research, largely explored through next-generation allergen vaccines. Properly induced trained immunity can shift allergic reactions toward a tolerogenic response [109]. Innate immune cells trained with specific stimuli, such as helminth-derived or certain bacterial- or yeast-derived products, can acquire anti-inflammatory features and display tolerance [35,110,111], which may have important implications for chronic inflammatory diseases, including allergies.

AIT is currently the only disease-modifying treatment for IgE-mediated allergic diseases. It involves the gradual administration of increasing doses of allergens over several years to induce tolerance to common allergens, including pollens, house dust mites (HDMs), insect venom, and foods [112,113,114]. The main components of AIT include allergens (e.g., pollen, house dust mites), allergen derivatives (e.g., recombinant allergens, peptides, hypoallergens), allergen extracts (e.g., dust mites, animal dander, insect venom), and adjuvants. Allergen derivatives can enhance immune tolerance, making AIT a safer and more effective treatment [115]. Common adjuvants include alum, calcium phosphate, microcrystalline tyrosine, and monophosphoryl lipid A (MPL, a TLR4 agonist). These adjuvants can promote Th1 responses, which help counteract Th2-mediated allergic reactions [116,117,118]. It is well known that allergic inflammation is driven by Th2 responses [119]. By promoting Th1 responses, these adjuvants can help counteract Th2-mediated reactions and support allergen tolerance.

The benefits of AIT include a reduced need for pharmacotherapy (e.g., inhaled corticosteroids), prevention of new sensitizations, and long-term clinical effects that persist after therapy completion. While the immunological effects of AIT have traditionally been attributed to adaptive immune modulation, emerging evidence indicates that innate immune reprogramming and trained immunity-like mechanisms also play a central role in the induction and maintenance of allergen tolerance [120,121].

Allergoid-mannan conjugates, a form of AIT, can reprogram monocytes into tolerogenic DCs through metabolic and epigenetic rewiring [122,123]. Allergoid-mannan conjugates enhance the expression of programmed death ligand-1 (PD-L1) and IL-10, while activating mTOR signaling, thereby promoting the differentiation of tolerogenic dendritic cells [123]. These tolerogenic DCs promote the expansion of regulatory T cells (Tregs) and suppress Th2-driven allergic inflammation.

Immune tolerance refers to the long-term unresponsiveness of the adaptive immune system to self-antigens or harmless non-self-antigens, governed by a complex interplay of genetic, epigenetic, and environmental factors [124,125,126]. Establishing long-term tolerance to allergens involves a complex network of interactions that modulate the functions of basophils, mast cells, allergen-specific regulatory T and B cells, and promote the production of allergen-specific antibodies [127]. AIT modulates IgE-FcεRI interactions and regulates cytokine signaling pathways, contributing to reduced allergic inflammation [128,129]. AIT induces the production of anti-allergic IgG4 and the selective depletion of IgE-producing B cells [128]. Because AIT can induce long-term immune tolerance, it has become a cornerstone in the management of allergic disorders [130].

ILCs can be classified into three functionally distinct subsets based on their cytokine production, surface markers, and transcription factor expression patterns: group 1 (ILC1), group 2 (ILC2), and group 3 (ILC3), which correspond functionally to Th1, Th2, and Th17 T cell subsets, respectively [131].

LCs reside in mucosal barriers and lymphoid tissues and contribute to the maintenance of immune tolerance in the gut [132,133]. ILC2s can suppress CD4^+^ and CD8^+^ T cell responses through IL-10 production, as demonstrated in humanized mouse models of graft-versus-host disease (GVHD) [132]. Sublingual immunotherapy (SLIT) induces IL-10-producing ILCs in patients with grass pollen allergies, contributing to the establishment of immune tolerance [134,135]. A recent study investigating mechanisms of AIT in grass pollen allergy shows that both KLRG1^+^ ILC2 precursors and mature prostaglandin D2 receptor (CRTH2^+^) Killer cell lectin-like receptor G1 (KLRG1^+^) ILC2s can produce the immunosuppressive cytokine IL-10, highlighting the role of ILC2 activation in successful AIT [136]. These findings indicate that activation of ILCs enhances the therapeutic efficacy of AIT by promoting immune tolerance.

*Pleurotus pulmonarius* (PP) crude extract, containing β-glucans, confers protection against Cryptococcus neoformans by inducing IL-10 secretion from monocyte-derived macrophages [137]. AIT-induced immune tolerance is maintained through complex interactions among the innate, adaptive, and humoral immune responses, prominently involving IL-10 in patients with allergic rhinitis (AR) [138].

### 6.2. AIT Suppresses Th2 Responses

Peptide-based allergen immunotherapy (PIT) prevents allergen-reactive peripheral/effector memory CD62L low Th2 cells from producing Th2 cytokines and confers protection against allergens such as pollens in a mouse model of allergic asthma [139]. AIT suppresses Th2 immune responses and reduces the expression of epithelial-derived alarmins, such as IL-25 and IL-33, in mouse models of allergic asthma [140,141]. AIT ameliorates airway hyperreactivity and restores epithelial integrity by suppressing IL-25–induced endoplasmic reticulum stress in allergic asthma models [142]. These reports indicate that AIT exerts anti-allergic effects by suppressing Th2 responses.

### 6.3. ILC3 Induced Immune Tolerance During AIT

ILC3s play key roles in oral tolerance by promoting the expansion and function of Treg cells [143]. ILC3s contribute to immune tolerance by creating a regulatory cytokine milieu, including TGF-β, that promotes Treg- mediated bystander suppression, leading to tolerance toward unrelated antigens [144]. Oral immunotherapy (OIT) activates ILC3s and is associated with hypomethylation of the FOXP3 gene in a murine model of cow’s milk allergy [145]. ILC3s induce immune tolerance in the gut by promoting the differentiation and maintenance of microbiota-specific RORγt^+^ regulatory T cells [146]. Macrophages, in response to food antigens, increase the production of IL-1β, which activates ILC3 [147]. ILC3-derived IL-2 is critical for maintaining FOXP3^+^ Tregs, preserving immunological homeostasis, and enforcing oral tolerance to food antigens in the small intestine [147]. IL-2 promotes tolerogenic FOXP3^+^ Tregs and NK cells to limit graft-versus-host disease in mouse models [148]. These findings suggest that ILC3s may contribute to the immunomodulatory effects of AIT in suppressing allergic inflammation.

### 6.4. AIT Promotes Immune Tolerance via IgG4

AIT in patients with IgE-mediated birch pollen allergy increases the production of birch pollen-specific IgG4, which contributes to the development of allergen tolerance [149]. IL-10 suppresses IgE production while promoting the generation of allergen-specific IgG4, which competes with IgE for allergen binding on basophils and mast cells, thereby inhibiting allergic responses [150,151]. IgE-allergen-IgG4 complexes can inhibit allergic effector cell activation by simultaneously engaging FcεRI and the inhibitory FcγRIIb receptor, thereby preventing degranulation and allergic responses [152]. SLIT increases allergen-specific IgG2 and IgG4 levels and expands the corresponding memory B-cell populations, while having little to no effect on the IgE memory B-cell compartment [153]. AIT-induced IgG4 and IgA antibodies contribute to immune tolerance by blocking allergen-IgE interactions and shifting the immune response away from Th2 toward Th1 and regulatory pathways [154].

### 6.5. AIT Induces Polarization of Macrophages

M1 macrophage polarization exerts an anti-allergic effect during allergic inflammation [79,119]. IgG4 induces a shift from M2a to tolerogenic M2b macrophages through cross-linking of the inhibitory receptor FcγRIIb [155]. M2b macrophages are characterized by the secretion of the chemokine CCL1, which binds to its receptor CCR8, as well as the cytokines IL-6, IL-10, and TNF-α [155]. AIT can increase the frequency of allergen-specific IgD-expressing B cells in patients sensitized to HDM, suggesting a possible role for IgD in the immunomodulatory effects of AIT [156]. Beekeepers tolerant to bee venom have higher serum levels of phospholipase A2-specific IgD, and children with milk or egg allergy undergoing oral immunotherapy (OIT) exhibit increased serum allergen-specific IgD (e.g., β-lactoglobulin and ovalbumin) compared with placebo controls [157]. Some studies suggest that elevations in allergen-specific IgD are associated with the presence of low-affinity IgE, a form of IgE that may induce oral tolerance [158].

### 6.6. AIT Induces Immune Tolerance via Tregs and Bregs

Successful AIT is associated with an increase in Bregs [157], FOXP3^+^ Tregs, with evidence also suggesting involvement of RORγt-expressing Treg subsets [159] and T follicular regulatory (T_FR_) cells [160].

Subcutaneous (SCIT) and sublingual (SLIT) grass pollen allergen immunotherapy induce changes in chromatin accessibility in circulating T follicular helper (cT_FH_) cells and T follicular regulatory (T_FR_) cells, shifting their epigenetic profiles toward those observed in non-atopic individuals [160]. SCIT and SLIT decrease IL-4 production and IL-21 expression by cT_FH_ cells [160]. Epicutaneous immunotherapy (EPIT) for food allergy promotes Treg responses and induces immune tolerance, associated with increased expression of IL-10 and TGF-β and decreased expression of the Th2 cytokines IL-4 and IL-5 [161]. IL-10–secreting Tregs specific for environmental allergens are predominant in healthy individuals [162]. In pediatric atopic patients and in mouse models of ovalbumin (OVA)-induced skin inflammation, IL-10 production is largely associated with activated, antigen-specific Tregs and Bregs [163,164]. Bregs contribute to immune tolerance through IL-10 production, which suppresses pro-inflammatory cytokine responses and promotes the development and function of Tregs [165,166]. Recent research has highlighted the importance of IL-10–producing Bregs and protective antibodies in AIT-induced long-term tolerance in patients with allergic respiratory diseases [167]. The role of human Bregs in immune tolerance is supported by the increased frequency of IL-10-producing Bregs observed in individuals undergoing AIT after repeated high-dose bee venom exposure [168].

### 6.7. AIT Induces Immune Tolerance via Immune Checkpoint Molecules

Immune tolerance can be induced by targeting antigen presentation or interfering with co-stimulatory pathways and Th2 cytokine production [169,170,171]. Allergen tolerance induced by AIT involves suppression of Th2 responses mediated by immune-suppressive molecules such as cytotoxic T lymphocyte antigen 4 (CTLA-4) and programmed death-1 (PD-1) [172]. Tregs-expressed CTLA-4 and PD-1, together with IL-10 and TGF-β, promote peripheral tolerance to egg allergens [173,174].

### 6.8. AIT Can Modulate Gut Microbiomes to Induce Immune Tolerance

The gut microbiota influences mucosal and systemic immune responses through interactions with immune cells in lymphoid tissues, including Peyer’s patches and other gut-associated lymphoid tissues (GALT). Probiotics can act as adjuvants to enhance the efficacy of AIT by decreasing the IgE and increasing Treg cells [175]. These findings suggest a protective role of the gut microbiome in AR. Allergen-specific SLIT improves symptoms of AR and is associated with increased abundance of *Streptococcus parasanguinis* [176]. These findings suggest that *Streptococcus parasanguinis* may serve as a potential microbial modulator or biomarker for predicting or improving SLIT efficacy. Strains of *Lactobacillus* promote immune tolerance by increasing Treg cells and anti-inflammatory cytokines such as IL-10 and TGF-β while suppressing Th2 cytokines, including IL-4 and IL-5 [161]. Gut commensal bacteria promote immune tolerance by inducing FOXP3^+^ Tregs and modulating IFN signaling pathways [177]. Short-chain fatty acids (SCFAs) produced by commensal gut bacteria promote a tolerogenic phenotype in DCs, leading to the induction of IL-10–secreting Tregs [178]. SCFAs increase the number of Tregs and enhance IL-10 production by Th1 cells, contributing to immune tolerance [179]. SCFAs, such as propionic acid, can enhance IL-10 production and support AIT–induced tolerance in AR [180]. AIT may influence the gut microbiome, potentially contributing to anti-allergic effects. Further studies are needed to investigate the impact of AIT on gut microbial composition and metabolites. Based on these reports, the administration of gut commensal bacteria may enhance the therapeutic efficacy of AIT. Future research directions include engineering probiotic bacteria, designing microbial consortia to induce immune tolerance, and developing precision AIT strategies mediated by gut commensal bacteria.

## 7. Discussion and Perspectives

Hematopoietic stem cells can transmit their trained immunity phenotype to their progeny, enabling the long-term persistence of trained immune responses that may last for weeks, months, or even years [181]. Parental immune experiences, such as pathogen exposure, can leave an immunological imprint that modifies innate immune responses in offspring, priming them to respond more effectively or differently upon subsequent encounters with the same pathogen [182]. This transgenerational transmission of trained immunity may be mediated by epigenetic mechanisms in gametes, including altered DNA methylation in sperm or oocytes [183]. Further investigation of transgenerational trained immunity is essential for a comprehensive understanding of chronic inflammatory diseases, including allergic disorders. Insights from this research may also inform the development of safer and more effective AIT vaccines.

During AIT, DCs exhibit a reduction in their Th2-promoting phenotype, including decreased expression of GATA-3, CD141, and receptor protein kinase 4 [184]. Accordingly, these Th2-associated markers may serve as potential biomarkers for predicting responsiveness to AIT. Complement component C1q and stabilin-1 (STAB1) have been proposed as early biomarkers of AIT efficacy [185,186]. Increased levels of secretoglobin 1A1 in the upper and lower airway secretions of patients with allergic rhinitis following AIT suggest that this molecule may act as a novel anti-inflammatory mediator contributing to long-term local immune regulation [187]. A single-center prospective study of house dust mite (HDM) sublingual immunotherapy (SLIT) identified six metabolites—lactic acid, ornithine, linolenic acid, creatinine, arachidonic acid, and sphingosine—that differed significantly between AIT responders and non-responders, suggesting their potential involvement in allergic inflammation and their value as indicators of treatment response [188].

Leukotriene A4 hydrolase has been found at higher levels in AIT responders compared with non-responders in patients with Artemisia pollen allergy, suggesting its potential as a predictive biomarker of treatment efficacy [189].

A deeper understanding of AIT-induced immune mechanisms is critical for validating biomarkers that can reliably predict treatment outcomes and enhance therapeutic success. Despite substantial advances in elucidating these mechanisms, clinically useful biomarkers for patient selection and treatment monitoring remain limited, underscoring the need for further discovery to enable safer and more cost-effective AIT.

Its allergen specificity and long-lasting effects make AIT an attractive therapeutic option for appropriately selected patients. However, the clinical efficacy of AIT depends on multiple factors, including the generation of tolerogenic immune responses, precise molecular diagnosis of causative allergens, and the availability of high-quality allergen formulations.

Although trained immunity can provide protection against recurrent and heterologous infections, its dysregulated activation in innate immune cells may contribute to chronic inflammation. To prevent such adverse outcomes during AIT, it is necessary to better characterize the immune and non-immune cell populations capable of developing trained immunity in response to different stimuli. Furthermore, the influence of host microbiota on trained innate immune responses should be further explored to elucidate the mechanisms underlying AIT-induced immune tolerance.

**Figure 5:**
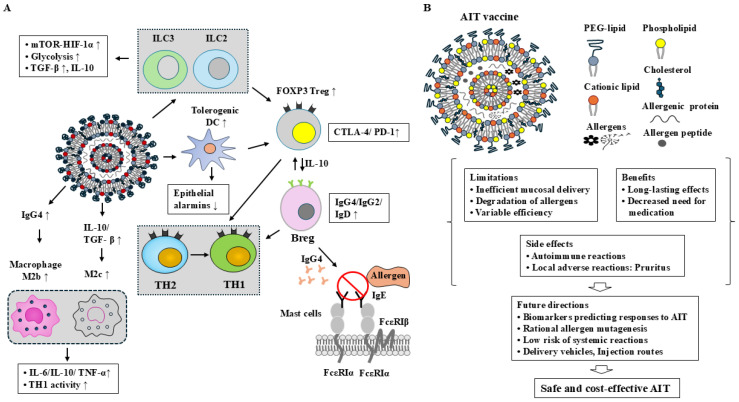
Mechanisms associated with AIT and future directions for developing safe and effective AIT vaccines. (**A**) AIT promotes the generation of tolerogenic DCs, which induce the differentiation of Tregs. AIT also induces Bregs, contributing to immune tolerance through the production of IgG4 and IgD. Furthermore, AIT promotes the generation of mature ILC2s that facilitate immune tolerance via IL-10 production. AIT-induced ILC3s enhance immune tolerance by increasing IL-2 production and expanding the Treg cell population. In addition, AIT promotes the differentiation of tolerogenic macrophages, including M2b and M2c subsets. Overall, AIT increases the number of FOXP3^+^ Treg cells and Bregs and upregulates the expression of tolerogenic molecules such as PD-L1, SOCS1, SOCS3, and IL-10. AIT-induced IgG4 shifts immune responses from a Th2- to a Th1-dominant profile and inhibits IgE binding to FcεRI. The thick arrow denotes the direction of reaction. ↑ denotes an increase in expression or activity. ↓ denotes an increase in expression. (**B**) The clinical benefits of AIT include reduced medication requirements, prevention of new allergic sensitizations, and long-lasting therapeutic effects. However, challenges remain, including the need for targeted delivery to innate immune cells, accumulation of long-term efficacy data, and further improvements in the stability, efficacy, and safety of AIT. The hollow arrow denotes the direction of reaction.

AIT is limited by challenges such as poor mucosal delivery, enzymatic degradation of allergens, and variable clinical efficacy. Subcutaneous immunotherapy (SCIT) for allergic rhinoconjunctivitis is generally safe, though autoimmune-like reactions have been reported [190], whereas sublingual immunotherapy (SLIT) for allergic asthma may cause local adverse effects, including throat irritation and oral pruritus [191]. Addressing these challenges is essential to enhancing the safety and efficacy of AIT vaccines. Rational allergen mutagenesis may promote trained immunity while reducing allergenicity. For rapid, short-term treatment, intralymphatic immunotherapy (ILIT), which involves direct injection of allergens into lymph nodes, represents a promising approach for efficiently inducing allergen-specific tolerance. Epicutaneous immunotherapy (EPIT) can deliver low doses of allergen while maintaining a low risk of systemic reactions. The mechanisms underlying AIT and future perspectives for developing safe and effective AIT vaccines are depicted (Figure 5).

## 8. Conclusions

Trained immunity represents a form of innate immune memory driven by metabolic and epigenetic reprogramming of innate immune cells. Growing evidence suggests that this process contributes to long-lasting immune regulation and may influence the development and treatment of allergic diseases. Therefore, targeting trained immunity may offer a promising strategy for improving long-term disease control beyond conventional symptomatic therapies. However, important questions remain regarding the duration, specificity, and safety of innate immune memory. Further studies are needed to determine how trained immunity can be effectively and safely harnessed for the prevention and treatment of allergic diseases.

Current challenges in AIT include the need for prolonged treatment duration (3–5 years), minimizing immune-related adverse effects, broadening the availability of allergen formulations, and developing personalized vaccines to maximize therapeutic efficacy. Although clinical trials of AIT vaccines have shown measurable clinical benefits [191], further studies incorporating modified allergens and novel adjuvants are warranted to improve both safety and efficacy. Continued interdisciplinary research integrating immunology, metabolism, epigenetics, and vaccine science will be critical for translating these advances into clinical practice. Current evidence suggests a promising future for AIT vaccines.

## Data Availability

No new data were created in this study. Data sharing is not applicable to this article.

## References

[1] Isachesku E., Cismaru A., Matzaraki V., Moorlag S.J.C.F.M., Mourits V.P., Koeken V.A.C.M., de Bree L.C.J., Joosten L.A.B., Berindan-Neagoe I., Netea M.G. (2025). The impact of interferon-γ pathway on trained immunity induction by vaccination with Bacille Calmette-Guérin. Sci. Rep..

[2] Righi S.E., Lilly E.A., Harriett A.J., Daly P.W., Jones M., Steele C., Noverr M.C., Fidel P.L. (2025). Induction of myelopoiesis by Candida dubliniensis drives protective trained immunity against sepsis in a Card9-dependent manner. mBio.

[3] Sánchez-Morales L., Porras N., Pérez-Domingo A., Pérez-Sancho M., García-Seco T., Diaz-Frutos M., Buendia A., Moreno I., Zamora L., Balseiro A. (2025). The impact of mycobacteria-induced trained immunity on SARS-CoV-2 vaccine responses. Front. Immunol..

[4] Kim H.G., Rincon J.C., Efron P.A., Maile R. (2025). DAMP-driven trained immunity: Metabolic and epigenetic reprogramming in critical illness and chronic inflammation. Front. Immunol..

[5] Risha M.A., Reddy K.D., Nemani S.P., Jakwerth C., Schmidt-Weber C., Bahmer T., Hansen G., von Mutius E., Rabe K.F., Dittrich A.M. (2024). Epigenetic training of human bronchial epithelium cells by repeated rhinovirus infections. Allergy.

[6] Brandi P., Conejero L., Cueto F.J., Martínez-Cano S., Dunphy G., Gómez M.J., Relaño C., Saz-Leal P., Enamorado M., Quintas A. (2022). Trained immunity induction by the inactivated mucosal vaccine MV130 protects against experimental viral respiratory infections. Cell Rep..

[7] Santecchia I., Vernel-Pauillac F., Rasid O., Quintin J., Gomes-Solecki M., Boneca I.G., Werts C. (2019). Innate immune memory through TLR2 and NOD2 contributes to the control of Leptospira interrogans infection. PLoS Pathog..

[8] Le K.T.T., Keur N., Middelkamp H., Linh Do T., van den Berg A., Orlova V., Joosten L.A.B., Netea M.G., Wijmenga C., Jonkers I. (2025). Cytokine-induced memory-like responses in endothelial cells link chronic inflammation to vascular disease risk. Mol. Omics.

[9] Boehme J.D., Jeron A., Schultz K., Melcher L., Schott K., Gelmez E., Kröger A., Stegemann-Koniszewski S., Bruder D. (2025). Epigenetic changes and serotype-specific responses of alveolar type II epithelial cells to Streptococcus pneumoniae in resolving influenza A virus infection. Cell Commun. Signal.

[10] Schneider R.F., Dubin A., Marten S.M., Roth O. (2024). Parent-Specific Transgenerational Immune Priming Enhances Offspring Defense-Unless Heat Stress Negates It All. Ecol. Evol..

[11] Jurado L.F., Daman A.W., Li Z., Ross V.M.S., Nikolaou K., Tran K.A., Loutochin O., McPherson V.A., Prével R., Tarancón R. (2025). A fungal-derived adjuvant amplifies the antitumoral potency of Bacillus Calmette-Guérin via reprogramming granulopoiesis. Immunity.

[12] Gong Y., Hao W., Xu L., Yang Y., Dong Z., Pan P., Bai Z., Huang J., Yang K., Jin Z. (2025). BCG-Derived Outer Membrane Vesicles Induce TLR2-Dependent Trained Immunity to Protect Against Polymicrobial Sepsis. Adv. Sci..

[13] Cirovic B., de Bree L.C.J., Groh L., Blok B.A., Chan J., van der Velden W.J.F.M., Bremmers M.E.J., van Crevel R., Händler K., Picelli S. (2020). BCG Vaccination in Humans Elicits Trained Immunity via the Hematopoietic Progenitor Compartment. Cell Host Microbe.

[14] Namgaladze D., Brüne B. (2023). Rapid glycolytic activation accompanying innate immune responses: Mechanisms andfunction. Front. Immunol..

[15] Divangahi M., Aaby P., Khader S.A., Barreiro L.B., Bekkering S., Chavakis T., van Crevel R., Curtis N., DiNardo A.R., Dominguez-Andres J. (2021). Trained immunity, tolerance, priming and differentiation: Distinct immunological processes. Nat. Immunol..

[16] Liu M., Shen X., Xu L. (2025). DNA methylation and histone modifications drive the trained immunity duration. Trends Immunol..

[17] Liu Y., Zhang H., Li Y., Zhao C. (2025). The effect of decitabine on human induced pluripotent stem cells (hiPSCs) derived CD34(+) cells expansion and the megakaryocytes generation and maturation. Am. J. Stem Cells.

[18] Murphy D.M., Mills K.H.G., Basdeo S.A. (2021). The Effects of Trained Innate Immunity on T Cell Responses; Clinical Implications and Knowledge Gaps for Future Research. Front. Immunol..

[19] Lim K.R.Q., Amrute J., Kovacs A., Diwan A., Williams D.L., Mann D.L. (2025). Toll-like receptor 4 induces trained innate immune tolerance in the heart in a model of stress-induced cardiomyopathy. Cardiovasc. Res..

[20] Hou X., Ding H., Wang H., Wang J., Liu H., Hu H., Billington C., Wang R., Zhang L. (2025). Gut bacteriophages induce specific-reprogramming of macrophages to generate a protective innate immunity response to lipopolysaccharide exposure. Gut Microbes.

[21] Cuenca-Escalona J., Kramer R., Marjalizo-Jimenez C., Domínguez-Andrés J., Netea M.G., Flórez-Grau G., de Vries I.J.M., Horrevorts S.K. (2026). BCG and beta-glucan primed monocytes yield dendritic cells that hamper the induction of pro-inflammatory T cell immunity. Cell Immunol..

[22] Wang H., Xie S., Huang S., Huang X., Zhang Y., Wu J., Fan X.Y., Hu Z. (2026). Mucosal Adenovirus-Vectored Rv2299c Vaccine Protects Against Tuberculosis by Inducing Trained Immunity in Dendritic Cells and Polyfunctional T Cells. Vaccines.

[23] Brockmann L., Tran A., Huang Y., Edwards M., Ronda C., Wang H.H., Ivanov I.I. (2023). Intestinal microbiota-specific Th17 cells possess regulatory properties and suppress effector T cells via c-MAF and IL-10. Immunity.

[24] Kurtz J., Andino R., Boraschi D., Contreras-Garduño J., Kachroo A., Khan I., Lanz Mendoza H., Mukherjee K., Peuß R., Ton J. (2025). Trained immunity and immune priming in plants and invertebrates. Elife.

[25] Damani-Yokota P., Khanna K.M. (2025). Innate immune memory: The evolving role of macrophages in therapy. Elife.

[26] Vuscan P., Kischkel B., Hatzioannou A., Markaki E., Sarlea A., Tintoré M., Cuñé J., Verginis P., de Lecea C., Chavakis T. (2024). Potent induction of trained immunity by *Saccharomyces cerevisiae* β-glucans. Front. Immunol..

[27] Sobén M., Guerrero P., Guiu A., Yáñez A., Gil M.L. (2025). Candida albicans-stimulated hematopoietic stem and progenitor cells generate trained neutrophils with enhanced mitochondrial ROS production that defend against infection. PLoS Pathog..

[28] Kleinnijenhuis J., Quintin J., Preijers F., Joosten L.A., Ifrim D.C., Saeed S., Jacobs C., van Loenhout J., de Jong D., Stunnenberg H.G. (2012). Bacille Calmette-Guerin induces NOD2-dependent nonspecific protection from reinfection via epigenetic reprogramming of monocytes. Proc. Natl. Acad. Sci. USA.

[29] Naqvi N., Ahuja Y., Zarin S., Alam A., Ali W., Shariq M., Hasnain S.E., Ehtesham N.Z. (2025). BCG’s role in strengthening immune responses: Implications for tuberculosis and comorbid diseases. Infect. Genet. Evol..

[30] Qi C., Liu Z., Kilic G., Sarlea A.S., Debisarun P.A., Liu X., Mekonnen Y.A., Li W., Grasshoff M., Alaswad A. (2025). Long-term DNA methylation changes mediate heterologous cytokine responses after BCG vaccination. Genome Biol..

[31] Rahman M.A., Goldfarbmuren K.C., Sarkis S., Bissa M., Gutowska A., Schifanella L., Moles R., Doster M.N., Andersen H., Jethmalani Y. (2025). BCG immunization mitigates SARS-CoV-2 replication in macaques via monocyte efferocytosis and neutrophil recruitment in lungs. JCI Insight.

[32] Al-Qahtani A.A., Alhamlan F.S., Al-Qahtani A.A. (2024). Pro-Inflammatory and Anti-Inflammatory Interleukins in Infectious Diseases: A Comprehensive Review. Trop. Med. Infect. Dis..

[33] Pal S., Rafiq Z., Kumari R., Al Aiyan A., Al-Ramadi B., Kishore U., Ponnachan P. (2025). Trained Innate Immunity. Adv. Exp. Med. Biol..

[34] Li Y., Chen M., Li J., Hu J. (2025). The Role of Pattern Recognition Receptors in Epigenetic and Metabolic Reprogramming: Insights into Trained Immunity. J. Inflamm. Res..

[35] Quintin J., Saeed S., Martens J.H.A., Giamarellos-Bourboulis E.J., Ifrim D.C., Logie C., Jacobs L., Jansen T., Kullberg B.J., Wijmenga C. (2012). Candida albicans infection affords protection against reinfection via functional reprogramming of monocytes. Cell Host Microbe.

[36] Zakeri A., Everts B., Williams A.R., Nejsum P. (2022). Antigens from the parasitic nematode Trichuris suis induce metabolic reprogramming and trained immunity to constrain inflammatory responses in macrophages. Cytokine.

[37] Chen Z., Yong T., Wei Z., Zhang X., Li X., Qin J., Li J., Hu J., Yang X., Gan L. (2024). Engineered Probiotic-Based Personalized Cancer Vaccine Potentiates Antitumor Immunity through Initiating Trained Immunity. Adv. Sci..

[38] González-Pérez M., Baranda J., Pérez-Rodríguez L., Conde P., Calle-Fabregat C., Berges-Buxeda M.J., Dimitrov A., Arranz J., Rius-Rocabert S., Zotta A. (2025). In vitro protocol demonstrating five functional steps of trained immunity in mice: Implications on biomarker discovery and translational research. Cell Rep..

[39] Towriss M., MacVicar B., Ciernia A.V. (2023). Modelling Microglial Innate Immune Memory In Vitro: Understanding the Role of Aerobic Glycolysis in Innate Immune Memory. Int. J. Mol. Sci..

[40] Jia Z., Niu L., Guo J., Wang J., Li H., Liu R., Liu N., Zhang S., Wang F., Ge J. (2025). Pathogen-derived peptidoglycan skeleton enhances innate immune defense against Staphylococcus aureus via mTOR-HIF-1alpha-HK2-mediated trained immunity. Microbiol. Res..

[41] Huang X., Wu J., Xu J., Wang H., Chen Z., Fan X.Y., Hu Z. (2025). Mycobacterium tuberculosis Antigen Rv1471 Induces Innate Immune Memory and Adaptive Immunity Against Infection. J. Infect. Dis..

[42] Wang T., Yang X., Ren W., Meng J., Yao X., Chu H., Yao R., Zhai M., Zeng Y. (2025). Integrating miRNA, mRNA, and Targeted Metabolomics Analyses to Explore the Regulatory Mechanism of Cardiac Remodeling in Yili Horses. Biology.

[43] Li F.L., Liu J.P., Bao R.X., Yan G., Feng X., Xu Y.P., Sun Y.P., Yan W., Ling Z.Q., Xiong Y. (2018). Acetylation accumulates PFKFB3 in cytoplasm to promote glycolysis and protects cells from cisplatin-induced apoptosis. Nat. Commun..

[44] He X., Xu Q., Qiu K., Tian Z., Sun D., Han J., Ma C., Ren X., Wang D., Wang J. (2025). Songorine protects cartilage in osteoarthritis by targeting PFKFB3 to disrupt the glycolysis-inflammation positive feedback loop. Phytomedicine.

[45] Tran B.T., Luna P.N., Cao R., Le D.T., Thatavarty A., Maneix L., Kain B.N., Koh S., Catic A., King K.Y. (2025). Rare epigenetic alterations are conserved across hematopoietic differentiation stages after mycobacterial infection. JCI Insight.

[46] Lee K.M., Kim K.H. (2025). Heat-inactivated Mycobacterium marinum as a vaccine adjuvant and a trained immunity inducer in rainbow trout (*Oncorhynchus mykiss*). Fish Shellfish Immunol..

[47] Liu J., Xiao X., Liao Y., Xu X., Liu Y., Tang A., Zeng X., Yang P. (2024). Allergen specific immunotherapy regulates macrophage property in the airways. Arch. Biochem. Biophys..

[48] Vierboom M.P.M., Dijkman K., Sombroek C.C., Hofman S.O., Boot C., Vervenne R.A.W., Haanstra K.G., van der Sande M., van Emst L., Domínguez-Andrés J. (2021). Stronger induction of trained immunity by mucosal BCG or MTBVAC vaccination compared to standard intradermal vaccination. Cell Rep. Med..

[49] Tulic M.K., Hodder M., Forsberg A., McCarthy S., Richman T., D’Vaz N., van den Biggelaar A.H., Thornton C.A., Prescott S.L. (2011). Differences in innate immune function between allergic and nonallergic children: New insights into immune ontogeny. J. Allergy Clin. Immunol..

[50] Neeland M.R., Koplin J.J., Dang T.D., Dharmage S.C., Tang M.L., Prescott S.L., Saffery R., Martino D.J., Allen K.J. (2018). Early life innate immune signatures of persistent food allergy. J. Allergy Clin. Immunol..

[51] Parnham M.J., Norris V., Kricker J.A. (2025). Promoting immune defensive responses of epithelial cells in airway disease. Front. Allergy.

[52] Charles N., Blank U. (2025). IgE-Mediated Activation of Mast Cells and Basophils in Health and Disease. Immunol. Rev..

[53] Ogulur I., Mitamura Y., Yazici D., Pat Y., Ardicli S., Li M., D’Avino P., Beha C., Babayev H., Zhao B. (2025). Type 2 immunity in allergic diseases. Cell. Mol. Immunol..

[54] Zou H., Duan J., Xie Y., Chen R., Ye Y., Zhang A., Yang P., Yang G., Liu X. (2025). Benzo[a]pyrene exacerbates allergen-induced airway inflammation through NLRP3-dependent dendritic cell activation and pathogenic T helper cell polarization. Front. Immunol..

[55] DeStefano S., Grychtol R., Funken D., Habener A., Tamm S., Stanke F., Greiner W., Beyer K., Hamelmann E., Kabesch M. (2025). NLRP3 regulates epithelial barrier integrity and protects from airway hyperresponsiveness in experimental allergic asthma. Front. Immunol..

[56] Mathä L., Romera-Hernández M., Steer C.A., Yin Y.H., Orangi M., Shim H., Chang C., Rossi F.M., Takei F. (2021). Migration of Lung Resident Group 2 Innate Lymphoid Cells Link Allergic Lung Inflammation and Liver Immunity. Front. Immunol..

[57] Topczewska P.M., Rompe Z.A., Jakob M.O., Stamm A., Leclère P.S., Preußer A., Duerr C.U., Thole L.M.L., Kotsch K., Artis D. (2023). ILC2 require cell-intrinsic ST2 signals to promote type 2 immune responses. Front. Immunol..

[58] Zou W., Ma D., Sun F., Chen Z., Chen Y., Li X., Chen M., Lin M., Shi H., Wu B. (2025). Maternal OM-85 administration alleviates offspring allergic airway inflammation by downregulating IL-33/ILC2 axis. Pediatr. Allergy Immunol..

[59] Sun H., Wu Y., Zhang Y., Ni B. (2021). IL-10-Producing ILCs: Molecular Mechanisms and Disease Relevance. Front. Immunol..

[60] Emami Fard N., Xiao M., Sehmi R. (2023). Regulatory ILC2-Role of IL-10 Producing ILC2 in Asthma. Cells.

[61] Alashkar Alhamwe B., Meulenbroek L.A.P.M., Veening-Griffioen D.H., Wehkamp T.M.D., Alhamdan F., Miethe S., Harb H., Hogenkamp A., Knippels L.M.J., Pogge von Strandmann E. (2020). Decreased Histone Acetylation Levels at Th1 and Regulatory Loci after Induction of Food Allergy. Nutrients.

[62] Bai S., Su X., Kong D., Feng C., Zhang X., Pan Y., Zhao J., Sun J., Li W. (2025). Selective HDAC8 inhibition by PCI-34051 attenuates inflammation and airway remodeling in asthma via miR-381-3p-TGFbeta3 axis. J. Transl. Int. Med..

[63] Penninger P., Brezovec H., Tsymala I., Teufl M., Phan-Canh T., Bitencourt T., Brinkmann M., Glaser W., Ellmeier W., Bonelli M. (2024). HDAC1 fine-tunes Th17 polarization in vivo to restrain tissue damage in fungal infections. Cell Rep..

[64] Jiang Q., Ding Y., Li F., Rosenfeld S., Pang Q., Geng X. (2025). Inhibition of class IIa HDACs reduces neuroinflammation via NEU1-LAMP1 regulation and promotes M2 macrophage polarization in ischemic stroke. Brain Res..

[65] Eom S., Kim Y., Park D., Lee H., Lee Y.S., Choe J., Kim Y.M., Jeoung D. (2014). Histone deacetylase-3 mediates positive feedback relationship between anaphylaxis and tumor metastasis. J. Biol. Chem..

[66] Wang Y., Wang H. (2022). The emerging role of histone deacetylase 1 in allergic diseases. Front. Immunol..

[67] Pavlenko E., Ruengeler T., Engel P., Poepsel S. (2022). Functions and Interactions of Mammalian KDM5 Demethylases. Front. Genet..

[68] Liu J., Liu Z., Geng X., Wu Y., Mo L., Liao Y., Liu Y., Yang P. (2025). KDM5A: A Master Epigenetic Regulator of Th2 Immunity and Allergic Disease Pathogenesis. Immunology.

[69] Ptaschinski C., Mukherjee S., Moore M.L., Albert M., Helin K., Kunkel S.L., Lukacs N.W. (2015). RSV-Induced H3K4 Demethylase KDM5B Leads to Regulation of Dendritic Cell-Derived Innate Cytokines and Exacerbates Pathogenesis In Vivo. PLoS Pathog..

[70] Audu C.O., Wolf S.J., Joshi A.D., Moon J.Y., Melvin W.J., Sharma S.B., Davis F.M., Obi A.T., Wasikowski R., Tsoi L.C. (2024). Histone demethylase JARID1C/KDM5C regulates Th17 cells by increasing IL-6 expression in diabetic plasmacytoid dendritic cells. JCI Insight.

[71] Song C., Wang G., Ma X., Mao P., Lu W., Zhang H., Liu L., Zhang Y., Li X. (2023). The effect of miR-155-5p on M1 polarization of Kupffer cells and immune response during liver transplantation through regulating the expression of KDM5D. Mol. Immunol..

[72] Tian Z., Zheng H., Fan Y., Li B., Huang Z., Wang M., Zhang J., Zhao J., Wang S., Xie J. (2025). Upregulated Hexokinase-2 in Airway Epithelium Regulates Apoptosis and Drives Inflammation in Asthma via Peptidylprolyl Isomerase F. Cells.

[73] Xuan L., Ren L., Zhang W., Du P., Li B., An Z. (2024). Formaldehyde aggravates airway inflammation through induction of glycolysis in an experimental model of asthma exacerbated by lipopolysaccharide. Sci. Total Environ..

[74] Zhou W., Zhang J., Chowdhury N.U., Norlander A.E., Toki S., Abney M., Rusznak M., Gibson-Corley K.N., Cook D.P., Newcomb D.C. (2025). PGI2 signaling metabolically reprograms CD4 Th2 cells and represses allergic airway inflammation. J. Immunol..

[75] Liu J., Li Y., Liu M., Li X., Liu C., Zhu Y. (2025). Formononetin ameliorates allergic asthma by inhibiting JUN and suppressing type immune responses mediated by ILC2 cells. Mol. Immunol..

[76] Ham J., Yang W., Kim H.Y. (2025). Tissue-Specific Metabolic Reprogramming in Innate Lymphoid Cells and Its Impact on Disease. Immune Netw..

[77] Zhang X., Liu J., Li X., Zheng G., Wang T., Sun H., Huang Z., He J., Qiu J., Zhao Z. (2025). Blocking the HIF-1α/glycolysis axis inhibits allergic airway inflammation by reducing ILC2 metabolism and function. Allergy.

[78] Peng X., Zhou P., Zhang K., Chen L., Tang M., Zhou Q., Peng J., Yang L. (2025). Deacetylation of TALDO1 by HDAC6 promotes glycolysis and nasopharyngeal carcinoma progression through a moonlighting function. Cell Death Dis..

[79] Lv L.X., Zhang P., Ma Y., Sun Y., Wang H., Zhao X.F., Wang J.X. (2025). Histone H3K27 acetylation mediated by KAT8 maintains antiviral trained immunity in shrimp induced by inactivated white spot syndrome virus. Commun. Biol..

[80] Abbring S., Wolf J., Ayechu-Muruzabal V., Diks M.A.P., Alhamwe B.A., Alhamdan F., Harb H., Renz H., Garn H., Garssen J. (2019). Raw Cow’s Milk Reduces Allergic Symptoms in a Murine Model for Food Allergy-A Potential Role For Epigenetic Modifications. Nutrients.

[81] Mijač S., Banić I., Genc A.M., Lipej M., Turkalj M. (2024). The Effects of Environmental Exposure on Epigenetic Modifications in Allergic Diseases. Medicina.

[82] Xu X.T., Fan J.K., Chen X., Que S.Y., Zhang R.X., Xie Y.Y., Zhou T.C., Ji K., Zhao Z.F., Chen J.J. (2025). Royal jelly acid inhibits NF-κB signaling by regulating H3 histone lactylation to alleviate IgE-mediated mast cell activation and allergic inflammation. Phytomedicine.

[83] Abel-Fernández E., Martínez M.J., Galán T., Pineda F. (2023). Going over Fungal Allergy: *Alternaria alternata* and Its Allergens. J. Fungi.

[84] Martín-Cruz L., Sevilla-Ortega C., Angelina A., Domínguez-Andrés J., Netea M.G., Subiza J.L., Palomares O. (2023). From trained immunity in allergy to trained immunity-based allergen vaccines. Clin. Exp. Allergy.

[85] Daman A.W., Antonelli A.C., Redelman-Sidi G., Paddock L., Khayat S., Ketavarapu M., Cheong J.G., Jurado L.F., Benjamin A., Jiang S. (2025). Microbial cancer immunotherapy reprograms hematopoiesis to enhance myeloid-driven anti-tumor immunity. Cancer Cell.

[86] Martín-Cruz L., Benito-Villalvilla C., Angelina A., Subiza J.L., Palomares O. (2024). Trained immunity-based vaccines for infections and allergic diseases. J. Allergy Clin. Immunol..

[87] Angulo M., Ramos-Vega A., Angulo C. (2025). Trained immunity-based Adjuvated vaccines (TIbAV) approach: Beta-glucans as example. Vaccine.

[88] Zhang J.G., Zhou C.K., Gao Y., Zhang X.M., Ma K., Peng Z.R., Luo X.Y., Liu Z.Z., Lin X.Q., Chen W. (2025). Trained immunity driven by Enterococcus faecalis ribosomal protein S11 enhances antigen presentation and boosts influenza vaccine efficacy via nanoparticle delivery. Int. J. Biol. Macromol..

[89] Tang X., Sun Y., Zhang J., Li H., Lu Y., Yuan L., Li H., Liu X., Song Y., Zhang Y. (2025). Host-pathogen hybrid glycocalyx mimicking lipid nanoparticles induce trained immunity as a promising platform for universal vaccines. J. Control. Release.

[90] Martín-Cruz L., Palomares O. (2025). Allergen-Specific Immunotherapy and Trained Immunity. Allergy.

[91] Arzola-Martínez L., Ptaschinski C., Lukacs N.W. (2023). Trained innate immunity, epigenetics, and food allergy. Front. Allergy.

[92] Khameneh H.J., Bolis M., Ventura P.M.O., Cassanmagnago G.A., Fischer B.A., Zenobi A., Guerra J., Buzzago I., Bernasconi M., Zaman G.J.R. (2024). The bacterial lysate OM-85 engages Toll-like receptors 2 and 4 triggering an immunomodulatory gene signature in human myeloid cells. Mucosal Immunol..

[93] Pittaluga-Villarreal J.R., Daniels C.M., Capece T., Kaplan P.R., Meier-Schellersheim M., Nita-Lazar A. (2025). PARP Inhibition Shifts Murine Myeloid Cells Toward a More Tolerogenic Profile in Vivo. Biomolecules.

[94] Muñoz-Wolf N., Lavelle E.C. (2021). Promotion of trained innate immunity by nanoparticles. Semin. Immunol..

[95] Amor N.P., Guo K., Zhang S., Xia J., Yang Y., Lin A. (2025). Lipid Nanoparticle: Beyond Delivery Vehicle-Unveiling Its Immunological Adjuvant Potential. FASEB J..

[96] Misra B., Hughes K.A., Pentz W.H., Surface M., Geldenhuys W.J., Bobbala S. (2025). TLR7-Adjuvanted Ionizable Lipid Nanoparticles for mRNA Vaccine Delivery. AAPS J..

[97] Muire P.J., Hanson L.A., Petrie-Hanson L. (2025). Rapid Natural Killer Cell Gene Responses, Generated by TLR Ligand-Induced Trained Immunity, Provide Protection to Bacterial Infection in *rag1^−/−^* Mutant Zebrafish (*Danio rerio*). Int. J. Mol. Sci..

[98] Gül S., Vergnaud J., Wang Q., Hery M., Mino J., Achour J., Domenichini S., Delomenie C., Perfettini J.L., Fay F. (2025). Deciphering the role of polyethylene glycol-lipid anchors in siRNA-LNP efficacy for P2y2 inhibition in bone marrow-derived macrophages. Int. J. Pharm..

[99] Swingle K.L., Hamilton A.G., Han X., Liao K.C., Safford H.C., Thatte A.S., Geisler H.C., Xu J., Saw T.Y., Wan Y. (2025). Circular RNA lipid nanoparticle vaccine against SARS-CoV-2. Proc. Natl. Acad. Sci. USA.

[100] Di L., Hong N.E., Pavlova O., Asase C., Lapping S., Switala L.E., Allio A.L.S., Nayak L., Maiseyeu A. (2025). Epigenetic metabolite lipid nanoparticles alleviate venous thrombosis via bone marrow reprogramming. Blood Vessel Thromb. Hemost..

[101] Glassman F.Y., Balu-Iyer S.V. (2018). Subcutaneous administration of Lyso-phosphatidylserine nanoparticles induces immunological tolerance towards Factor VIII in a Hemophilia A mouse model. Int. J. Pharm..

[102] Rademacker S., Pinto Carneiro S., Molbay M., Catapano F., Forné I., Imhof A., Wibel R., Heidecke C., Hölig P., Merkel O.M. (2025). The impact of lipid compositions on siRNA and mRNA lipid nanoparticle performance for pulmonary delivery. Eur. J. Pharm. Sci..

[103] Baranov M.V., Kumar M., Sacanna S., Thutupalli S., van den Bogaart G. (2021). Modulation of Immune Responses by Particle Size and Shape. Front. Immunol..

[104] Kolbe T., Gaspard P., Mognetti B.M. (2025). Understanding Influenza A Virus Particles Detaching from Reconstructed Cell Surfaces. Nano Lett..

[105] Simpson S.R., Middleton D.D., Lukesh N.R., Islam M.J., Ehrenzeller S.A., Bachelder E.M., Ainslie K.M. (2024). Microparticles incorporating dual apoptotic factors to inhibit inflammatory effects in macrophages. J. Pharm. Sci..

[106] Chak V., Ovalle E.J., Bard J., Wohlfert E.A., Kay J.G., Balu-Iyer S. (2025). Single-cell transcriptional analysis of murine mesenteric lymph nodes following oral lyso-phosphatidylserine nanoparticle administration reveals cellular heterogeneity in tolerance features. J. Pharm. Sci..

[107] Nguyen N.H., Chen M., Chak V., Balu-Iyer S.V. (2022). Biophysical Characterization of Tolerogenic Lipid-Based Nanoparticles Containing Phosphatidylcholine and Lysophosphatidylserine. J. Pharm. Sci..

[108] Lai Y., Yang X., Wei D., Wang X., Sun R., Li Y., Ji P., Bao Y., Chu T., Zhang C. (2025). BCG-trained macrophages couple LDLR upregulation to type I IFN responses and antiviral immunity. Cell Rep..

[109] Montin D., Ottaviano G., Sangerardi M., Sgrulletti M., Chini L., Dellepiane R.M., Martire B., Rizzo C., Moschese V. (2022). Novel artful applications of vaccines at the horizon. Pediatr. Allergy Immunol..

[110] Mulindwa J., Lujumba I., Musiime C., Namulondo J., Kimuda M.P., Nyangiri O., Cuu G., Mwubaha C., Tukwasibwe S., Ssemaganda A. (2025). High Schistosoma mansoni infection intensity is associated with distinct gut microbiota and low levels of systemic cytokines in children along the Albert-Nile, Northern Uganda. BMC Microbiol..

[111] Moosavi M., Brødsgaard Kjærup R., Papanikolaou K., Wattrang E., Sørensen Dalgaard T. (2025). Indications of trained innate immunity by Escherichia coli vaccination or chitin feed supplementation assessed during Ascaridia galli infection in chickens. Mol. Immunol..

[112] Bachert C., Hoogeveen H., Ten Have R., Yu D., Worm M., Pfaar O., Jutel M., Distler A., Bozek A., Opstelten D.J. (2025). Subcutaneous Allergen Immunotherapy in Adults Allergic to House Dust Mites: A Phase 3 Randomized Controlled Trial. Allergy.

[113] Delgado J., Cárdenas R., Gelis S., Domínguez-Ortega J. (2025). Allergen immunotherapy for the control of moderate to severe allergic asthma: An evidence-based conjoint analysis to define candidate patient profiles in Spain and Portugal. Front. Allergy.

[114] Lee J.Z.X., Sit J.K.C., Leung N.Y.H., Chu K.H., Leung P.S.C., Leung T.F., Wai C.Y.Y. (2025). Next-Generation Allergen-Specific Immunotherapy for **Food** Allergy. Clin. Rev. Allergy Immunol..

[115] da Silva E.S., Fernandes A.M.S., Silva R.C., de Souza L.M., Sousa J.E.A., Orrico-Ferreira C.M., Alcântara-Neves N.M., Pacheco L.G.C., Pinheiro C.D.S. (2025). Long-Term Administration of BTH2 Hypoallergenic Vaccine Candidate Induces Hallmarks of Allergen Immunotherapy in Murine Model of Blomia tropicalis-Induced Asthma. Biomedicines.

[116] Pourkamalzadeh M., Abtahi Froushani S.M., Ownagh A. (2025). Adjuvant Synergy: Alum and chlorogenic acid enhance Th1 responses and survival in a Salmonella typhimurium challenge model. Biologicals.

[117] Noori Goodarzi N., Barzi S.M., Ajdary S., Chiani M., Yekaninejad M.S., Badmasti F., Pourmand M.R. (2025). Immunogenic evaluation of LptD + LtgC as a bivalent vaccine candidate against Neisseria gonorrhoeae. J. Transl. Med..

[118] Jia S., Zhou X., Fang L., Jiang Z., De X., Liu R., Wang F., Ge J. (2025). Biomimetic mineralization material RMCP enhanced immunogenicity of subunit vaccine against Clostridium perfringens infection in mice. Int. J. Biol. Macromol..

[119] Kim M., Jo H., Kwon Y., Jeong M.S., Jung H.S., Kim Y., Jeoung D. (2021). MiR-154-5p-MCP1 Axis Regulates Allergic Inflammation by Mediating Cellular Interactions. Front. Immunol..

[120] Tong Y., Wang L., Wang L., Song J., Fan J., Lai C., Bao J., Weng C., Wang Y., Shuai J. (2024). Allergen immunotherapy combined with Notch pathway inhibitors improves HDM-induced allergic airway inflammation and inhibits ILC2 activation. Front. Immunol..

[121] Zhou X., Simonin E.M., Jung Y.S., Galli S.J., Nadeau K.C. (2024). Role of allergen immunotherapy and biologics in allergic diseases. Curr. Opin. Immunol..

[122] Benito-Villalvilla C., Pérez-Diego M., Subiza J.L., Palomares O. (2022). Allergoid-mannan conjugates imprint tolerogenic features in human macrophages. Allergy.

[123] Benito-Villalvilla C., Soria I., Pérez-Diego M., Fernández-Caldas E., Subiza J.L., Palomares O. (2020). Alum impairs tolerogenic properties induced by allergoid-mannan conjugates inhibiting mTOR and metabolic reprogramming in human DCs. Allergy.

[124] Duan S., Jia Z., Zheng L., Wu Y., Xu Z., Peng H., Xue J. (2025). Research advances on epigenetic modifications in dendritic cells in allergic rhinitis. Front. Immunol..

[125] Ashley S.E., Tang M.L.K. (2026). Modulating the Allergen-Specific Immune Response to Achieve Remission of Food Allergy Through Oral Immunotherapy. Adv. Exp. Med. Biol..

[126] Han X., Skatova V., Mikelov A., Ji X., Hoh R.A., Lee J.Y., Cao S., Seastedt H., Schuetz J., Fernandes A. (2025). Peanut allergy oral immunotherapy drives single-cell multi-omic changes in peanut-reactive T cells associated with sustained unresponsiveness. Nat. Immunol..

[127] Hsu P.S., Barnes E.H., Barnes M., McArthur M., Valerio C., Santner-Nanan B., Pinget G., Wang Y., Tran C.D., Lai C.L. (2025). Oral Peanut Immunotherapy with Butyrate Adjuvant (OPIA) in Children: A Randomised, Controlled Trial. Clin. Exp. Allergy.

[128] Tamaș T.P., Ciurariu E. (2025). Allergen Immunotherapy: Pitfalls, Perks and Unexpected Allies. Int. J. Mol. Sci..

[129] Shi L.L., Xiong P., Yang M., Ardicli O., Schneider S.R., Funch A.B., Kiykim A., Lopez J., Akdis C.A., Akdis M. (2025). Role of IgG4 Antibodies in Human Health and Disease. Cells.

[130] Asiri A., Alzahrani F., Ismail G.M., Almonawar N., Althubaiti S., Alkhalifah W., Almutairi O.M., Albeladi R., Alsaqri R.A., Alzhrani L.M. (2025). Efficacy and Safety of Sublingual and Subcutaneous Immunotherapy in Children with Allergic Rhinitis: A Systematic Review of Randomized Trials Including Direct and Indirect Comparisons. Int. J. Gen. Med..

[131] Simoni Y., Newell E.W. (2018). Dissecting human ILC heterogeneity: More than just three subsets. Immunology.

[132] Reid K.T., Colpitts S.J., Mathews J.A., Santos Carreira A., Murphy J.M., Borovsky D.T., Jegatheeswaran S., Cui W., Alfaro Moya T., Sachewsky N. (2025). Cell therapy with human IL-10-producing ILC2s limits xenogeneic graft-versus-host disease by inhibiting pathogenic T cell responses. Cell Rep..

[133] Parrón-Ballesteros J., Gordo R.G., López-Rodríguez J.C., Olmo N., Villalba M., Batanero E., Turnay J. (2023). Beyond allergic progression: From molecules to microbes as barrier modulators in the gut-lung axis functionality. Front. Allergy.

[134] Bohle B., Kinaciyan T., Gerstmayr M., Radakovics A., Jahn-Schmid B., Ebner C. (2007). Sublingual immunotherapy induces IL-10-producing T regulatory cells, allergen-specific T-cell tolerance, and immune deviation. J. Allergy Clin. Immunol..

[135] Schulten V., Tripple V., Aasbjerg K., Backer V., Lund G., Würtzen P.A., Sette A., Peters B. (2016). Distinct modulation of allergic T cell responses by subcutaneous vs. sublingual allergen-specific immunotherapy. Clin. Exp. Allergy.

[136] Golebski K., Layhadi J.A., Sahiner U., Steveling-Klein E.H., Lenormand M.M., Li R.C.Y., Bal S.M., Heesters B.A., Vilà-Nadal G., Hunewald O. (2021). Induction of IL-10-producing type 2 innate lymphoid cells by allergen immunotherapy is associated with clinical response. Immunity.

[137] Arromsava A., Chuchawankul S., Worasilchai N., Angkasekwinai P., Amornsupak K. (2025). Immunomodulatory activity of Pleurotus pulmonarius crude extract to human monocyte against Cryptococcus neoformans. BMC Complement Med. Ther..

[138] Huo Z., Gu J., Wu J., Wang C. (2025). Gut microbiome composition and its impact on response to allergen immunotherapy in adult patients with allergic rhinitis. Acta Microbiol. Immunol. Hung..

[139] Mackenzie K.J., Nowakowska D.J., Leech M.D., McFarlane A.J., Wilson C., Fitch P.M., O’Connor R.A., Howie S.E., Schwarze J., Anderton S.M. (2014). Effector and central memory T helper 2 cells respond differently to peptide immunotherapy. Proc. Natl. Acad. Sci. USA.

[140] Baker J.R., Rasky A.J., Landers J.J., Janczak K.W., Totten T.D., Lukacs N.W., O’Konek J.J. (2021). Intranasal delivery of allergen in a nanoemulsion adjuvant inhibits allergen-specific reactions in mouse models of allergic airway disease. Clin. Exp. Allergy.

[141] Wang T., Chi J., Li Z., Zhang Y., Wang Y., Ding M., Zhou B., Gui J., Li Q. (2024). Recombinant Art v4.01 protein produces immunological tolerance by subcutaneous immunotherapy in a wormwood pollen-driven allergic asthma female mouse model. PLoS ONE.

[142] Pfaar O., Fritzsching B., Wolf H., Woehlk C., Wüstenberg E. (2023). How does allergen immunotherapy-induced tolerance improve the airway epithelial barrier function: A mechanistical-driven hypothesis. Allergy.

[143] Rodrigues P.F., Wu S., Trsan T., Panda S.K., Fachi J.L., Liu Y., Du S., de Oliveira S., Antonova A.U., Khantakova D. (2025). Rorγt-positive dendritic cells are required for the induction of peripheral regulatory T cells in response to oral antigens. Cell.

[144] Bumbacea R.S., Boustani R., Panaitescu C., Haidar L., Buzan M.R., Bumbacea D., Laculiceanu A., Cojanu C., Spanu D., Agache I. (2022). Mechanisms of allergen immunotherapy supporting its disease-modifying effect. Immunotherapy.

[145] Vonk M.M., Diks M.A.P., Wagenaar L., Smit J.J., Pieters R.H.H., Garssen J., van Esch B.C.A.M., Knippels L.M.J. (2017). Improved Efficacy of Oral Immunotherapy Using Non-Digestible Oligosaccharides in a Murine Cow’s Milk Allergy Model: A Potential Role for Foxp3+ Regulatory T Cells. Front. Immunol..

[146] Lyu M., Suzuki H., Kang L., Gaspal F., Zhou W., Goc J., Zhou L., Zhou J., Zhang W., JRI Live Cell Bank (2022). ILC3s select microbiota-specific regulatory T cells to establish tolerance in the gut. Nature.

[147] Zhou L., Chu C., Teng F., Bessman N.J., Goc J., Santosa E.K., Putzel G.G., Kabata H., Kelsen J.R., Baldassano R.N. (2019). Innate lymphoid cells support regulatory T cells in the intestine through interleukin-2. Nature.

[148] Horwitz D.A., Kim D., Kang C., Brion K., Bickerton S., La Cava A. (2025). CD2-targeted nanoparticles encapsulating IL-2 induce tolerogenic Tregs and TGF-beta-producing NK cells that stabilize Tregs for long-term therapeutic efficacy in immune-mediated disorders. Front. Immunol..

[149] Baum C., Möbs C., Pfützner W. (2025). Booster Injection with Birch Pollen Extract Activates B-Cellular Memory Responses in Patients Having Completed Allergen Immunotherapy. Eur. J. Immunol..

[150] Clement R.L., Dilollo J., Rodríguez-López E.M., Guerrier C.M., Hill D.A. (2025). IFNγ Signaling Impairs Regulatory B Cell Function Resulting in Worse Control of Esophageal Food Allergy. Allergy.

[151] Cevhertas L., Ma S., Stanic B., Ochsner U., Jansen K., Ferstl R., Frei R., Chijioke O., Münz C., Zhang L. (2021). IL-10 induces IgG4 production in NOD-scid Il2rγ^null^ mice humanized by engraftment of peripheral blood mononuclear cells. Allergy.

[152] Qin L., Tang L.F., Cheng L., Wang H.Y. (2022). The clinical significance of allergen-specific IgG4 in allergic diseases. Front. Immunol..

[153] Heeringa J.J., McKenzie C.I., Varese N., Hew M., Bakx A.T.C.M., Aui P.M., Rolland J.M., O’Hehir R.E., van Zelm M.C. (2020). Induction of IgG_2_ and IgG_4_ B-cell memory following sublingual immunotherapy for ryegrass pollen allergy. Allergy.

[154] Garcia-Carmona Y., Curotto de Lafaille M.A. (2025). Advances in Food Allergy Immunotherapy: Current Strategies and Role of Antibodies Isotypes. Cells.

[155] Bianchini R., Roth-Walter F., Ohradanova-Repic A., Flicker S., Hufnagl K., Fischer M.B., Stockinger H., Jensen-Jarolim E. (2019). IgG4 drives M2a macrophages to a regulatory M2b-like phenotype: Potential implication in immune tolerance. Allergy.

[156] Boonpiyathad T., Pradubpongsa P., Mitthamsiri W., Satitsuksanoa P., Jacquet A., Sangasapaviliya A. (2020). Allergen-specific immunotherapy boosts allergen-specific IgD production in house dust mite-sensitized asthmatic patients. Allergy.

[157] Satitsuksanoa P., van de Veen W., Tan G., Lopez J.F., Wirz O., Jansen K., Sokolowska M., Mirer D., Globinska A., Boonpiyathad T. (2025). Allergen-specific B cell responses in oral immunotherapy-induced desensitization, remission, and natural outgrowth in cow’s milk allergy. Allergy.

[158] Itoh N., Ohshima Y. (2023). The dual aspects of IgD in the development of tolerance and the pathogenesis of allergic diseases. Allergol. Int..

[159] Li D., Cruz I., Sorkhabi S., Foley P.L., Wagner J., Bellanti J.A. (2025). Dose-response studies of methylated and nonmethylated CpG ODNs from Bifidobacterium longum subsp. infantis for optimizing Treg cell stimulation. Allergy Asthma Proc..

[160] Sharif H., Acharya S., Dhondalay G.K.R., Varricchi G., Krasner-Macleod S., Laisuan W., Switzer A., Lenormand M., Kashe E., Parkin R.V. (2021). Altered chromatin landscape in circulating T follicular helper and regulatory cells following grass pollen subcutaneous and sublingual immunotherapy. J. Allergy Clin. Immunol..

[161] Mennini M., Piccirillo M., Furio S., Valitutti F., Ferretti A., Strisciuglio C., De Filippo M., Parisi P., Peroni D.G., Di Nardo G. (2024). Probiotics and other adjuvants in allergen-specific immunotherapy for food allergy: A comprehensive review. Front. Allergy.

[162] Wang Z., Shahzad K.A., Li X., Cai B., Xu M., Li J., Tan F. (2024). Immunomodulatory effect of mesenchymal stem cells-derived extracellular vesicles to modulate the regulatory T cells and Th1/Th2 imbalance in peripheral blood mononuclear cells of patients with allergic rhinitis. Scand. J. Immunol..

[163] Gao X., Hou Z., Li X., Ouyang T., Yu S., Wang Y., Zhang G., Luo Y., He X., Liao W. (2025). Iron Deficiency Drives Th2-Mediated Immunity in Pediatric Atopic Dermatitis Through DNA Hypermethylation and TIGIT Suppression in IL-10-Producing Breg Cells. J. Asthma Allergy.

[164] Li D., Cruz I., Peltak S.N., Foley P.L., Bellanti J.A. (2025). Methylated CpG ODNs from *Bifidobacterium longum* subsp. *infantis* Modulate Treg Induction and Suppress Allergic Response in a Murine Model. Int. J. Mol. Sci..

[165] Lv Z., Wang T.Y., Bi Y., Li D., Wu Q., Wang B., Ma Y. (2025). BAFF overexpression in triple-negative breast cancer promotes tumor growth by inducing IL-10-secreting regulatory B cells that suppress anti-tumor T cell responses. Breast Cancer Res. Treat..

[166] Zhou M., Wen Z., Cheng F., Ma J., Li W., Ren H., Sheng Y., Dong H., Lu L., Hu H.M. (2016). Tumor-released autophagosomes induce IL-10-producing B cells with suppressive activity on T lymphocytes via TLR2-MyD88-NF-kappaB signal pathway. Oncoimmunology.

[167] Shigehara K., Kamekura R., Ikegami I., Sakamoto H., Yanagi M., Kamiya S., Kodama K., Asai Y., Miyajima S., Nishikiori H. (2023). Circulating T follicular helper 2 cells, T follicular regulatory cells and regulatory B cells are effective biomarkers for predicting the response to house dust mite sublingual immunotherapy in patients with allergic respiratory diseases. Front. Immunol..

[168] Boonpiyathad T., Meyer N., Moniuszko M., Sokolowska M., Eljaszewicz A., Wirz O.F., Tomasiak-Lozowska M.M., Bodzenta-Lukaszyk A., Ruxrungtham K., van de Veen W. (2017). High-dose bee venom exposure induces similar tolerogenic B-cell responses in allergic patients and healthy beekeepers. Allergy.

[169] Wu W., Ding F., Li Y., Fu Z. (2025). CXCL14 increase dendritic cell antigen presentation and promote asthma immune response. Pediatr. Discov..

[170] Nemoto Y., Morikawa R., Yonemoto Y., Tanaka S., Takei Y., Oshima S., Nagaishi T., Tsuchiya K., Nakamura T., Takenaka K. (2025). Intestinal CD4^-^CD8αβ^-^TCRαβ^+^ T cells function as tolerogenic antigen presenting cells in mice. Nat. Commun..

[171] Glamočlija S., Sabljić L., Schmid A., Radulović N., Gruden-Movsesijan A., Vasilev S., Inić-Kanada A., Wiedermann U., Schabussova I., Kosanović M. (2025). Trichinella spiralis-derived extracellular vesicles induce regulatory T cells and reduce airway allergy in mice. Front. Immunol..

[172] Jie X., Wang D., Da H., Li H., Zhao H., He J., Liu J., Ma Y., Qiang Z., Li Z. (2024). Increased inhibitory surface marker PD-1 expression in CD4^+^T cells and Th2^+^T cells in allergen-specific immunotherapy. Immunobiology.

[173] De Vlieger L., Nuyttens L., Keppens C., Ieven T., Matton C., Diels M., Verelst S., Raes M., Leus J., Coppens K. (2025). Egg allergen-specific T-cell and cytokine responses in healthy and egg-allergic children naturally tolerating baked egg. Pediatr. Allergy Immunol..

[174] Layhadi J.A., Starchenka S., De Kam P.J., Palmer E., Patel N., Keane S.T., Hikmawati P., Drazdauskaite G., Wu L.Y.D., Filipaviciute P. (2025). Ara h 2-expressing cucumber mosaic virus-like particle (VLP Peanut) induces in vitro tolerogenic cellular responses in peanut-allergic individuals. J. Allergy Clin. Immunol..

[175] Lamminpää I., Niccolai E., Amedei A. (2024). Probiotics as adjuvants to mitigate adverse reactions and enhance effectiveness in Food Allergy Immunotherapy. Scand. J. Immunol..

[176] Wu J., Wang D., He W.J., Li J.Y., Mo X., Li Y.J. (2024). Allergen-specific sublingual immunotherapy altered gut microbiota in patients with allergic rhinitis. Front. Cell Infect. Microbiol..

[177] Vasquez Ayala A., Hsu C.Y., Oles R.E., Matsuo K., Loomis L.R., Buzun E., Carrillo Terrazas M., Gerner R.R., Lu H.H., Kim S. (2024). Commensal bacteria promote type I interferon signaling to maintain immune tolerance in mice. J. Exp. Med..

[178] Kaisar M.M.M., Pelgrom L.R., van der Ham A.J., Yazdanbakhsh M., Everts B. (2017). Butyrate Conditions Human Dendritic Cells to Prime Type 1 Regulatory T Cells via both Histone Deacetylase Inhibition and G Protein-Coupled Receptor 109A Signaling. Front. Immunol..

[179] Saadh M.J., Allela O.Q.B., Ballal S., Mahdi M.S., Chahar M., Verma R., Al-Hussein R.K.A., Adil M., Jawad M.J., Al-Nuaimi A.M.A. (2025). The effects of microbiota-derived short-chain fatty acids on T lymphocytes: From autoimmune diseases to cancer. Semin. Oncol..

[180] Zhou C.J., Xie B.L., Han H.Y., Wang Y., Wang Y.H., Hong J.Y., Wei Y.X., Liu Z.G., Feng Y., Yang G. (2021). Short-Chain Fatty Acids Promote Immunotherapy by Modulating Immune Regulatory Property in B Cells. J. Immunol. Res..

[181] Gu J., Liu Q., Zhang J., Xu S. (2023). COVID-19 and trained immunity: The inflammatory burden of long covid. Front. Immunol..

[182] Dulfer E.A., Domínguez-Andrés J. (2024). Mechanisms involved in the transmission of trained immunity to offspring. J. Allergy Clin. Immunol..

[183] Cruz-Carrillo G., Camacho-Morales A. (2021). Metabolic Flexibility Assists Reprograming of Central and Peripheral Innate Immunity During Neurodevelopment. Mol. Neurobiol..

[184] Eljaszewicz A., Ruchti F., Radzikowska U., Globinska A., Boonpiyathad T., Gschwend A., Morita H., Helbling A., Arasi S., Kahlert H. (2021). Trained immunity and tolerance in innate lymphoid cells, monocytes, and dendritic cells during allergen-specific immunotherapy. J Allergy Clin. Immunol..

[185] Zimmer A., Bouley J., Le Mignon M., Pliquet E., Horiot S., Turfkruyer M., Baron-Bodo V., Horak F., Nony E., Louise A. (2012). A regulatory dendritic cell signature correlates with the clinical efficacy of allergen-specific sublingual immunotherapy. J. Allergy Clin. Immunol..

[186] Tan T.J., Delgado-Dolset M.I., Escribese M.M., Barber D., Layhadi J.A., Shamji M.H. (2022). Biomarkers of AIT: Models of prediction of efficacy. Allergol. Select.

[187] Zissler U.M., Jakwerth C.A., Guerth F., Lewitan L., Rothkirch S., Davidovic M., Ulrich M., Oelsner M., Garn H., Schmidt-Weber C.B. (2021). Allergen-specific immunotherapy induces the suppressive secretoglobin 1A1 in cells of the lower airways. Allergy.

[188] Xie S., Jiang S., Zhang H., Wang F., Liu Y., She Y., Jing Q., Gao K., Fan R., Xie S. (2021). Prediction of sublingual immunotherapy efficacy in allergic rhinitis by serum metabolomics analysis. Int. Immunopharmacol..

[189] Ma T.T., Cao M.D., Yu R.L., Shi H.Y., Yan W.J., Liu J.G., Pan C., Sun J., Wei Q.Y., Wang D.Y. (2020). Leukotriene A_4_ Hydrolase Is a Candidate Predictive Biomarker for Successful Allergen Immunotherapy. Front. Immunol..

[190] Thakor Philipp A., Anderson L. (2025). The Possible Unveiling of Myasthenia Gravis via Allergy Shots. Cureus.

[191] Roberts G., Just J., Nolte H., Hels O.H., Emeryk A., Vidal C. (2025). SQ House Dust Mite Sublingual Immunotherapy Tablet in Children with Allergic Asthma: A Randomised Phase III Trial. Allergy.

